# Global overview of suicidal behavior and associated risk factors among people living with human immunodeficiency virus: A scoping review

**DOI:** 10.1371/journal.pone.0269489

**Published:** 2023-03-20

**Authors:** Yi-Tseng Tsai, Sriyani Padmalatha K. M., Han-Chang Ku, Yi-Lin Wu, Nai-Ying Ko

**Affiliations:** 1 Department of Nursing, An Nan Hospital, China Medical University, Tainan, Taiwan; 2 Department of Nursing, College of Medicine, National Cheng Kung University, Tainan, Taiwan; 3 Operating Room Department, National Hospital of Sri Lanka, Colombo, Sri Lanka; 4 Department of Public Health, College of Medicine, National Cheng Kung University, Tainan, Taiwan; Rutland Regional Medical Center, UNITED STATES

## Abstract

Death by suicide is a major public health problem. People living with human immunodeficiency virus (PLHIV) have higher risk of suicidal behavior than the general population. The aim of this review is to summarize suicidal behavior, associated risk factors, and risk populations among PLHIV. Research studies in six databases from January 1, 1988, to July 8, 2021, were searched using keywords that included “HIV,” “suicide,” and “risk factors.” The study design, suicide measurement techniques, risk factors, and study findings were extracted. A total of 193 studies were included. We found that the Americas, Europe, and Asia have the highest rates of suicidal behavior. Suicide risk factors include demographic factors, mental illness, and physiological, psychological, and social support. Depression is the most common risk factor for PLHIV, with suicidal ideation and attempt risk. Drug overdosage is the main cause of suicide death. In conclusion, the current study found that PLHIV had experienced a high level of suicidal status. This review provides an overview of suicidal behavior and its risk factors in PLHIV with the goal of better managing these factors and thus preventing death due to suicide.

## Introduction

Death due to suicide is a major public health problem worldwide. According to the World Health Organization (WHO), approximately 700,000 people died worldwide due to suicide every year (an average of one death every 40 s) [1]. Suicide is a global phenomenon and can occur at any age. Acquired immunodeficiency syndrome (HIV) and human immunodeficiency virus (AIDS) is also a common public health issue, and currently there are more than 37.9 million people living with HIV/AIDS around the world [2]. The rate of suicide deaths in People living with HIV (PLHIV) is 100-fold higher than the rate that has been reported in the general population [3]. Prevalence estimates of suicidal ideation, attempts, and plans among people living with HIV/AIDS were more common and serious than those in the general population [4]. Suicide attempt rates among PLHIV with mental disorders and psychiatric treatment have continued to increase from the pre-highly active antiretroviral therapy (Pre-HAART) era (1988–1995) to the HAART era (1996–2008) from 27.8% to 35.1%, respectively [5].

Suicidal behavior is complex, with different levels of severity, ranging from suicidal ideation to suicide attempts and ultimately to the end of life by death due to suicide. Suicidal ideation is defined as thoughts, considerations, or plans to die by suicide, whereas suicide attempts are defined as failed attempts to die by suicide where the person survives [6–8]. That suicidal ideation is more common than suicide attempt and death by suicide, and the presence of suicidal ideation increases the risk of suicide attempt and death by suicide. The suggest a complex interrelationship between behavior and suicide attempts [8, 9]. Suicidal ideation is an important predictor of subsequent suicide attempts and dying by suicide [9, 10].

Suicidal behavior is a complicated process that ranges in degree of severity, from thinking about killing oneself (i.e., suicidal ideation) to doing it (i.e., suicide attempt and death by suicide). In the current study provided insights into the relationships among HARRT, depression, and suicidal status in PLHIV and evidence that depression played a mediating role in the association between suicide ideation and attempt. However, the relationship between these three-suicide behavior is unclear; for example, relationship between HARRT, and death by suicide or depression, and suicide attempts, therefore, this study will be a better feasibility to understanding relationship between these three-suicide behavior and could help prevent suicidal behavior in PLHIV, in whom suicide is a significant public health problem of HIV-infected adults. It is important to categorization of suicidal behaviors among PLHIV due to lack of overview of scope reviewing in this population, even suicide became significant life-threatening event in PLHIV [9].

In general, primary research studies consider only one or two suicidal behavior, such as only suicidal ideation or attempts, or both suicidal ideation and suicide attempts, within a single center or country. Some studies included specific at-risk populations like perinatal women, homosexual men, and prisoners with HIV. However, these studies did not involve all at-risk populations and their risk behavior [11–16]. In previous primary research, among PLHIV, poor social support, HIV stigma, mental disorders, and associated comorbidities were associated with increased suicide rates. Improvements in antiretroviral therapy have led to better survival rates in PLHIV; however, suicidal behavior remain a major health issue [17, 18].

Thus, exploring suicidal behavior using a wide range of global research studies is vital for primary healthcare professionals to plan early recognition of this and suicide prevention strategies. The aim of this review is to provide an overview of the rates of suicidal behavior and associated suicide risk factors among PLHIV. Detection of suicidal ideation and suicide attempts is important in planning early suicide prevention and optimizing HIV/AIDS management.

## Materials and methods

This review has been registered in the International Platform of Registered Systematic Review and Meta-analysis Protocols (INPLASY, Reg No: INPLASY202170033).

### Search strategy

Literature was searched using the following six databases: Embase, Ovid MEDLINE, CENTRAL, Web of Science, Academic Search Complete, and Psychology & Behavioral Sciences Collection. This was done after meeting with a public health librarian (the author CJF) and two members of the research team to clarify goals and further define the selection criteria to develop the literature search strategy. This review included studies published between January 1, 1988 and July 8, 2021 based on the Preferred Reporting Items for Systematic Reviews and Meta-Analyses guidelines [19].

English synonyms such as AIDS, T-lymphotropic virus, or human T-cell lymphotropic virus, type III human T-cell leukemia virus, type III lymphadenopathy-associated virus, LAV-HTLV-III, HTLV-III-LAV, type III infection, or HTLV-III infection were used in each database to identify suicidal behavior among PLHIV. We also used several control phrases from the Emtree and Medical Subject Headings (MeSH) databases. For Emtree, these included “Human immunodeficiency virus,” “Human immunodeficiency virus infection,” “suicidal behavior,” or “automutilation,” and “suicide,” and for MeSH, they included “HIV infections,” “HIV,” or “self-injurious behavior.” We supplemented the search results with the EndNote X9 bibliographical database, and the search results were manually screened, including the reference lists of relevant articles and previous systematic reviews to confirm the sensitivity of the search strategy (S1 File) [20].

### Eligibility criteria

The inclusion criteria were as follows: (1) the studies provided primary data on the prevalence or incidence of suicidal ideation, suicide attempts, or suicides measured using validated assessment tools or coded medical report data within a population-based study; (2) the participants were aged ≥15 years; (3) the participants were diagnosed with HIV/AIDS; and (4) the report was an original, published article in English or Chinese. The following types of studies were excluded: (1) the study population did not include PLHIV; (2) those unrelated to suicide; and (3) case report and review studies.

Titles and abstracts were independently screened by three researchers based on the inclusion and exclusion criteria after automatically removing duplicates using EndNote X9. Then, the full text of the selected studies was reviewed independently by three researchers, with any disagreement resolved by a fourth researcher to avoid selection bias. Disagreements regarding article inclusion were resolved by discussion between all authors.

### Quality assessment

All eligible studies were assessed for quality of evidence using the Joanna Briggs Institute Critical Appraisal Checklist for Prevalence Studies Scale, which contains nine items and four responses (yes, no, unclear, and not applicable) [21]. Studies with a total score of 8 and above were considered to have high quality evidence and were included in this systematic review. Study quality and risk of bias were independently assessed by three researchers, with any disagreements resolved by a fourth researcher.

### Data extraction

The authors YT and HC conceptualized the study and developed the research protocol. YT created the initial draft of the data extraction chart containing the study characteristics of interest *a priori*. YT, HC, and YL identified articles for full-text review. Full data extraction was then carried out independently on each article by YT, HC, and YL, and any disagreements were resolved through discussion with a fourth author. Data extraction was recorded on a standardized Excel sheet. The following details were listed: the name of the authors and the publication year, the name of the journal, country, setting, study design, sample size, included risk factors for suicide, available measurement tools, and prevalence of suicide ideation, suicide attempts, and completed suicide (Table 1).

**Table 1:** Study characteristic of selected 193 studies.

Author, Year of publication	Journal	Type of study	WHO region	Country	Sample size	Study setting	Measurement tool	Suicide ideation rate	Suicide attempt rate	Completed suicide rate	Risk factor
Marzuk, P. M. et al. (1988) [23]	Journal of the American Medical Association	retrospective study	Region of the Americas	United States	3,828	Database	N/A	N/A	N/A	680.56 per 100,000 person-years.	Age 25–59.
Perry, S. et al. (1990) [24]	Journal of the American Medical Association	cross-sectional study	Region of the Americas	United States	301	Hospital	Beck Depression Inventory (BDI).	28.6% (n = 86)	N/A	N/A	Depression.
Cote, T. R. et al. (1992) [25]	Journal of the American Medical Association	cohort study	Region of the Americas	United States	98,473 person-years.	Database	N/A	N/A	N/A	167 per 100,000 person-years.	Drug poisoning, firearms, suffocation, jumping from high places, age, race.
Gala, C. et al. (1992) [119]	Acta Psychiatrica Scandinavica	cross-sectional study	European region	Italy	213	Hospital	N/A	N/A	N/A	N/A	DSH, psychiatric history.
McKegney, F. P. et al. (1992) [26]	American Journal of Psychiatry	cross-sectional study	Region of the Americas	United States	404	Hospital	N/A	N/A	N/A	N/A	Organic mental disorders.
Rajs, J. et al. (1992) [77]	Acta Psychiatrica Scandinavica	retrospective study	European region	Sweden	85	Hospital	N/A	N/A	N/A	25% (n = 21)	Homo- and bisexual males, intravenous drug addicts, medicinal drug overdosage, low psychosocial support.
Brown, G. R. et al. (1993) [27]	Vaccine	longitudinal study	Region of the Americas	United States	394	Hospital	Standard anxiety (HARS) and depression (HDRS) rating scale scores.	17% (n = 67)	N/A	N/A	Anxiety, depression, major mood disorder, psychoactive substance use disorder, medication overdose, cutting wrists, firearms and jumping. violent.
Chu, S. Y. et al. (1993) [195]	American Journal of Public Health	retrospective study	Region of the Americas	United States	19,564	Database	N/A	N/A	N/A	0.3% (n = 58)	HIV exposure category and time between diagnosis and death.
Twiname, B. G. (1993) [187]	Journal of the Association of Nurses in AIDS Care	cross-sectional study	Regionof the Americas	United States	80	Hospital	Beck Depression Inventory (BDI).	32.5% (n = 26)	N/A	N/A	Depression
Alfonso, C. A. et al. (1994) [28]	Psychosomatics	retrospective study	Region of the Americas	United States	2,363	Hospital	N/A	N/A	21.8% (n = 515)	N/A	HIV seropositivity.
Craven, D. E. Et al. (1994) [160]	Annals of Internal Medicine	retrospective study	Region of the Americas	United States	7	Hospital	N/A	N/A	42.8% (n = 3)	N/A	CD4 cell count, depression, history of illicit narcotic use.
Hanvelt, R. A. et al. (1994) [122]	AIDS	cohort study	Region of the Americas	Canada	144,876	Database	N/A	N/A	N/A	9.4% (n = 13,618)	Indirect cost of production lost
Van Haastrecht, H. J. A. Et al. (1994) [29]	AIDS	cohort study	European region	Netherlands	86	Hospital	N/A	N/A	N/A	8.13% (n = 7)	Overdose, intravenous drug-using (IDU)
Carvajal, M. J. et al. (1995) [78]	AIDS Care	retrospective study	European region	Spain	422	Hospital	N/A	1.18% (n = 5)	4.02% (n = 17)	0.47% (n = 2)	Accidental overdose, IDU.
Mancoske, R. J. et al. (1995) [30]	Social Work	cohort study	Region of the Americas	United States	51	Database	N/A	N/A	N/A	175 per 10,000-person year	Nonmetropolitan areas.
Sherr, L. (1995) [79]	AIDS Care	retrospective study	European region	United Kingdom	188	Clinic	N/A	50.5% (n = 95)	21.4% (n = 40)	0.5% (n = 1)	Overdosing, diagnosis with peaks at or around diagnosis.
Bindels, P. J. et al. (1996) [31]	Lancet	Retrospective study	European region	Netherlands	131	Hospital	N/A	N/A	N/A	13% (n = 17)	End stage illness.
Breitbart, W. et al. (1996) [188]	American Journal of Psychiatry	cross-sectional study	Region of the Americas	United States	378	Database	N/A	55% (n = 207)	N/A	N/A	Euthanasia, physician- assisted suicide (PAS).
Dannenberg, A. L. Et al. (1996) [32]	Journal of the American Medical Association	cohort study	Region of the Americas	United States	4,147	Database	N/A	N/A	N/A	49 per 100000 person-years.	Physician- assisted suicide (PAS), depression, hopelessness, psychological distress, social factors.
Marzuk, P. M et al. (1997) [33]	American Journal of Psychiatry	cross-sectional study	Region of the Americas	United States	1,511	Database	N/A	N/A	N/A	8.67% (n = 131)	Male, time from screening to death was less than 3 months.
Rosengard, C. et al. (1997) [34]	AIDS Care	cross-sectional study	Region of the Americas	United States	86	Database	N/A	12.78% (n = 11)	N/A	N/A	Bereavement, feeling burdened, low social support, subjective social integration.
Sherr, L. et al. (1997) [80]	Genitourinary Medicine	retrospective study	European region	United Kingdom	100	Clinic	N/A	69% (n = 69)	31% (n = 31)	N/A	Female.
Wood, K. A. et al. (1997) [35]	AIDS Care	cross-sectional study	Region of theAmericas	United States	50	Hospital	N/A	60% (n = 30)	N/A	N/A	Schizophrenia.
Gil, F. et al. (1998) [36]	AIDS Patient Care STDS	cross-sectional study	Region of the Americas	United States	91	Hospital	Suicidal ideation (Scale for Suicide Ideation Self-Report).	63.4%(n = 57)	N/A	N/A	Psychological symptoms, physical symptoms, social support, satisfaction with the social support received, poor sexual adjustment.
Kelly, B. et al. (1998) [37]	Psychosomatics	cross-sectional study	Region of the Americas	United States	164	Hospital	Beck Depression Inventory and the General Health Questionnaire (28-item version).	N/A	21% (n = 34)	N/A	Male, psychiatric disorder, neuroticism, unemployment.
Swartz, H. A. et al. (1998) [38]	Psychiatric Services	cross-sectional study	Region of the Americas	United States	33	Hospital	The 24-item Hamilton Rating Scale for Depression (Ham-D). Beck Depression Inventory (BDI).	N/A	52% (n = 17)	N/A	Substance abuse.
Kalichman, S. C. et al. (2000) [39]	Psychiatric Services	cross-sectional study	Region of the Americas	United States	113	Clinic	Beck Depression Inventory (BDI)	26% (n = 29)	N/A	N/A	Men, whites, gay.
Malbergier, A. et al. (2001) [194]	AIDS Care	cross-sectional study	Western Pacific region	Australia	30	Hospital	N/A	N/A	27% (n = 8)	N/A	Depression.
Bonnet, F. et al. (2002) [81]	HIV Medicine	retrospective study	European region	France	107	Hospital	N/A	N/A	N/A	6% (n = 7)	
Cohen, M. H. et al. (2002) [40]	American Journal of Medicine	cohort study	Region of the Americas	Brazil	1,902	Database	N/A	N/A	N/A	(n = 10)	Women, depression, IDU with hepatitis C infection, smoking, age.
Heckman, T. G. Et al. (2002) [41]	Ann Behav Med	cross-sectional study	Region of the Americas	United States	201	Clinic	N/A	38%(n = 76)	N/A	N/A	Depression, less coping self-efficacy, transmitting their HIV infection to others, stress associated with AIDS-related stigma.
Lochet, P. et al. (2003) [82]	HIV Medicine	cross-sectional study	European region	France	174	Hospital	General Health question (GHD-28).	13.2% (n = 23)	N/A	N/A	N/A
Roy, A. (2003) [42]	Acta Psychiatrica Scandinavica	cross-sectional study	Region of the Americas	United States	149	Clinic	The depression section of the Structured Clinical Interview for DSM-IV.	N/A	44.3% (n = 66)	N/A	Female, younger, substance dependence, depression.
May, T. et al. (2003) [83]	Presse Medicale	retrospective study	European region	France	864	Database	N/A	N/A	N/A	11% (n = 6)	N/A
Summers, J. et al. (2004) [43]	Death Studies	cross-sectional study	Region of the Americas	United States	93	Clinic	Hamilton Depression Rating Scale. Suicide Assessment Questions from the National Institute of Mental Health Diagnostic Interview Schedule Version III-A (DIS).	51.61% (n = 48)	25.8% (n = 24)	N/A	N/A
Cooperman, N. A. et al. (2005) [15]	Journal of Behavioral Medicine	cross-sectional study	Region of the Americas	United States	207	Clinic	Suicidal Ideation and Behavior Suicidal ideation was assessed with a scale.	N/A	26% (n = 54)	N/A	Women, having children, employed.
Gielen, A. C. Et al. (2005) [44]	Womens Health Issues	cross-sectional study	Region of the Americas	United States	310	Clinic	Intimate partner violence. IPV screening tool widely used in health care settings.	31% (n = 96)	16% (n = 49)	N/A	Abused women, anxiety, depression.
Krentz, H. B. Et al. (2005) [15]	HIV Medicine	retrospective study	Region of the Americas	Canada	560	Hospital	N/A	N/A	N/A	7% (n = 40)	N/A
Lewden, C. et al. (2005) [84]	International Journal of Epidemiology	Retrospective study	Region of the Americas	United States	964	Hospital	N/A	N/A	N/A	4% (n = 38)	HIV infection had been diagnosed recently, smoking, alcohol.
Olley, B. O. et al. (2005) [123]	AIDS Care	cross-sectional study	African region	South Africa	149	Hospital	The MINI International Neuropsychiatric Interview (MINI).	N/A	54% (n = 12)	N/A	Posttraumatic stress disorder.
Petrushkin, H. et al. (2005) [124]	Psychiatric Bulletin	cross-sectional study	African region	Uganda	46	Hospital	The MINI International Neuropsychiatric Interview (MINI).	N/A	17.39% (n = 8)	N/A	Depression.
Jin, H. et al. (2006) [85]	Journal of Affective Disorders	cross-sectional study	European region	France	28	Hospital	Beck Depression Inventory-I (BDI).	18% (n = 4)	N/A	N/A	Depression.
Lu, T. H. et al. (2006) [161]	Journal of the Formosan Medical Association	cohort study	Western Pacific Region	China	752	Hospital	The MINI International Neuropsychiatric Interview (MINI).	N/A	N/A	4.8% (n = 14)	HAART.
Olley, B. O. (2006) [125]	African Journal of AIDS Research	cross-sectional study	African region	South Africa	105	Hospital	The MINI International Neuropsychiatric Interview (MINI).	11.4% (n = 12)	N/A	N/A	Depression.
Robertson, K. et al. (2006) [162]	Death Stud	cross-sectional study	Western Pacific Region	Taiwan	191	Hospital	The MMPI-2 is a well standardized self-report instrument consisting of 13 basic scales.	39.27% (n = 75)	26.70% (n = 51)	N/A	N/A
Shelton, A. J. et al. (2006) [45]	AIDS Care	cross-sectional study	Region of the Americas	United States	54	Clinic	A semi-structured interview; Brief Symptom Inventory (BSI; Derogatis & Spencer, 1982). The MMPI-2 is a well standardized self-report instrument consisting of 13 basic scales. Ten clinical scales (Hypochondriasis, Depression, Hysteria, Psychopathic Deviate, Masculinity-Femininity, Paranoia, Psychasthenia, Schizophrenia, Hypomania, Social Introversion).	59.26% (n = 32)	29.63 (n = 16)	N/A	White.
Carrico, A. W. et al. (2007) [46]	Aids	cross-sectional study	Region of the Americas	United States	2,909	Clinic	N/A	19% (n = 553)	N/A	N/A	Marijuana use, depression.
Lewden, C. et al. (2008) [86]	Jaids-Journal of Acquired Immune Deficiency Syndromes	cohort study	Region of the Americas	United States	1,042	Hospital	Perceived social support the 24-item Social Provisions Scale (Cronbach’s a = 0.82). Coping self-efficacy Coping self-efficacy was assessed with an abbreviated (15-item) version of a 26-item scale (Cronbach’s a = 0.92).	N/A	N/A	5% (n = 52)	Male, CD4 cell count.
Lifson, A. R. et al. (2008) [87]	HIV Clinical Trials	case-controlled study	Region of the Americas	United States	11,593	Hospital	N/A	N/A	N/A	0.2% (n = 23)	N/A
Preau, M. et al. (2008) [120]	AIDS Care	cross-sectional study	European region	France	2,932	Hospital	Depression symptoms and suicidal ideation the 21-item Beck Depression Inventory (BDI) (Cronbach’s a = 0.86).	N/A	22% (n = 645)	N/A	Born in France, female, younger adults, lower level of education, unemployed, difficult household financial situation.
Quintana-Ortiz, R. A. Et al. (2008) [121]	Ethn Dis	cohort study	European region	France	714	Database	N/A	N/A	22% (n = 157)	N/A	Male, HIV/AIDS status at study entry, IDU, stress factors related to filial relationships, psychoactive substance, isolation, depression, anxiety.
Sherr, L. et al. (2008) [47]	AIDS Care	cross-sectional study	Region of the Americas	United States	778	Clinic	N/A	31% (n = 241)	N/A	N/A	Heterosexual man, black, white, unemployment, lack of disclosure of HIV status, stopped antiretroviral treatment, physical symptoms, psychological symptoms, poorer quality of life.
Yang, C. H. et al. (2008) [163]	HIV Medicine	retrospective study	Western Pacific Region	Taiwan	1,161	Hospital	N/A	N/A	N/A	5.5% (n = 62)	
Lawler, K. et al. (2009) [126]	AIDS and Behavior	cross-sectional study	African region	Africa	120	Hospital	Beck Depression Inventory-Fast Screen for Medical Patients (BDI-FS). Mood Module (MM) of the Primary Care Evaluation of Mental Disorders (Prime-MD)	12% (n = 15)	N/A	N/A	Depression.
Shacham, E. et al. (2009) [48]	AIDS Patient Care & STDs	cross-sectional study	Region of the Americas	United States	514	Clinic	The Patient Health Questionnaire (PHQ-9).	15% (n = 78)	N/A	N/A	unemployed.
Hessamfar-Bonarek, M. Et al. (2010) [88]	International Journal of Epidemiology	retrospective study	European region	United Kingdom	1,013	Database	N/A	N/A	N/A	women 4% (n = 40) men 9% (n = 91)	Female, poor socio-economic conditions.
Keiser, O. et al. (2010) [5]	American Journal of Psychiatry	cohort study	European region	Switzerland	15,275	Database	N/A	N/A	N/A	150 died by suicide (rate 158.4 per 100,000 person-years).	Older patients, male, IDU, patients with advanced clinical stage of HIV illness.
Lampe, F. C. et al. (2010) [89]	Journal of Acquired Immune Deficiency Syndromes	cross-sectional study	European region	United Kingdom	188	Clinic	N/A	N/A	27% (n = 51)	N/A	Depression, anxiety.
Lau, J. T. et al. (2010) [164]	AIDS Care	cross-sectional study	Western Pacific Region	China	176	Clinic	Depression, Anxiety, and Stress Scales (Cronbach’s 0.80 to 0.83).	34% (n = 60)	8% (n = 14)	N/A	Depression, anxiety, stress.
Lawrence, S. T., et al. (2010) [10]	Clinical Infectious Diseases	cohort study	Region of the Americas	United States	1,216	Clinic	The psycho-social domains assessed by the PROs include depression (PHQ-9).	14% (n = 170)	N/A	N/A	Male, white, young middle-aged, substance abuse, depression.
Peng, E. Y. et al. (2010) [165]	AIDS Care	cross-sectional study	Western Pacific Region	Taiwan	535	Prisons	The five-item Brief Symptom Rating Scale (BSRS-5).	12.5% (n = 67)	(n = 22)	N/A	Depression, anxiety, psychological distress.
Peng, E. Y. C. Et al. (2010) [166]	Journal of the Formosan Medical Association	cross-sectional study	Western Pacific Region	Taiwan	479	Prisons	The five-item Brief Symptom Rating Scale (BSRS-5).	N/A	4.2% (n = 20)	N/A	Psychiatric morbidity, physical pain or discomfort, depression, anxiety.
Schlebusch, L. et al. (2010) [127]	African Journal of Psychiatry	cohort study	African region	South Africa	112	Hospital	Comprehensive mental state examination and administration of a semi structured questionnaire to obtain biographical, socio-demographic and other relevant data.	N/A	67.2 per 100, 000 person-years.	N/A	N/A
Aldaz, P. et al. (2011) [90]	Bmc Public Health	cohort study	European region	Spain	1145	Database	N/A	N/A	N/A	(n = 7)	IDU.
Atkinson, J. H. et al. (2011) [167]	Journal of Affective Disorders	longitudinal study	Western Pacific Region	China	203	Clinic	World Mental Health Composite International Diagnostic Interview (WMH-CIDI, version 3.0).	49.26% (n = 100)	N/A	N/A	Depression.
Cejas M, R. et al. (2011) [92]	Journal of Psychosomatic Research	cross-sectional study	European region	Romania	125	Hospital	Calgary Depression Scale. Plutchick Suicide Risk Scale.	12.1% (n = 66)	N/A	N/A	Abuse drug, psychiatric disorder, previous suicide attempts, CD4 levels (poor immune status).
Davis, S. J. et al. (2011) [49]	Journal of Rehabilitation	cross-sectional study	Region of the Americas	United States	71	Clinic	Suicidal/Homicidal Thought Scale. Depression Symptom Scale.	5.7% (n = 4)	N/A	N/A	N/A
Halman, M. et al. (2011) [50]	Canadian Journal of Infectious Diseases and Medical Microbiology	Retrospective study	Region of the Americas	Canada	87	Palliative care center	Modified HIV Stressor Scale. Social Support Scale.	9.2% (n = 8)	10.3% (n = 9)	N/A	Substance abuse
Kinyanda, E. et al. (2011) [128]	BMC Psychiatry	cohort study	African region	Uganda	618	Clinic	N/A	N/A	5.99% (n = 37)	N/A	Female, family history of mental illness, negative coping style, alcohol dependency disorder, food insecurity, Stress.
Lee, B. et al. (2011) [168]	Journal of the International Association of Physicians in AIDS Care	case-control study	South-East Asian region	Thailand	219	Hospital	N/A	N/A	N/A	15.5% (n = 34)	Depression.
Badiee, J. et al. (2012) [20]	Journal of Affective Disorders	cross-sectional study	Region of the Americas	United States	1,560	Clinic	Stress Score index’ 5. Social support index’ Cronbach of 0.84. M.I.N.I. neuropsychiatric interview (MINI Plus).	26% (n = 405)	13% (n = 204)	N/A	Substance abuse, depression, mood disruption.
Capron, D.W. et al. (2012) [51]	AIDS Patient Care STDS	cross-sectional study	Region of the Americas	United States	164	Database	Children’s Depression Inventory (CDI).	N/A	N/A	N/A	Negative affectivity.
Chikezie, U. E. et al. (2012) [129]	AIDS Care	cross-sectional study	African region	Nigeria	150	Hospital	Beck Depression Inventory-II (BDI-II).	34.7% (n = 52)	9.3% (n = 14)	N/A	Older age, female, unemployed, single, no children, living alone, comorbid illness, partner who was also with the disease.
Ellis, J. et al. (2012) [93]	HIV Medicine	retrospective study	European Region	United Kingdom	46	Clinic	N/A	N/A	13% (n = 6)	N/A	Alcohol misuse, recreational drug use, mental health problems.
Govender, R. D. et al. (2012) [130]	South African Journal of Psychiatry	cross-sectional study	African region	South Africa	157	Hospital	Positive and Negative Affect Scale (PANAS) (range of alpha coefficients: 0.85 to 0.93). Anxiety Sensitivity Index-3 (ASI-3) (Cronbach a = 0.95).	17.1% (n = 27).	N/A	N/A	Depression.
Govender, R. D. et al. (2012) [131]	South African Journal of Psychiatry	cross-sectional study	African region	South Africa	109	Hospital	Beck Hopelessness Scale (BHS). Beck Depression Inventory (BDI).	N/A	N/A	N/A	N/A
Jia, C. X. et al. (2012) [94]	The Journal of Clinical Psychiatry	cohort study	European Region	Denmark	9,900	Database	Beck Depression Inventory (BDI-II).	N/A	N/A	38.6% (n = 3,821)	Single people, low income, psychiatric illness, first time recently, were treated as inpatients, had a recent hospital contact, had multiple hospital contacts because of the illness.
Kinyanda, E. et al. (2012)[132]	BMC Psychiatry	cross-sectional study	African region	Uganda	618	Clinic	Beck Hopelessness Scale (BHS). Beck Depression Inventory (BDI).	N/A	3.9% (n = 24)	N/A	Female, negative life events, previous psychiatric history, major Depression disorder.
Lewis, E. L. et al. (2012) [133]	Health Care for Women International	cross-sectional study	Region of the Americas	United States	62	Hospital	Beck Depression Inventory-Fast Screen (BDI-FS). Mood Module (MM) of the Primary Care Evaluation of Mental Disorders (Prime-MD).	11% (n = 7)	N/A	N/A	Female.
Protopopescu, C. et al. (2012) [95]	Antiviral Therapy	cohort study	European Region	France	1,095	Database	N/A	1.28 (n = 14)	N/A	0.46 (n = 5)	N/A
Schlebusch, L. et al. (2012) [134]	International Journal of Environmental Research and Public Health	cross-sectional study	African region	South Africa	189	Hospital	N/A	24.2% (n = 46)	N/A	N/A	Young age, male.
Sherr, L. et al. (2012) [96]	Women & Health	cross-sectional study	European Region	United Kingdom	262	Clinic	Health-Related Quality of Life (HRQOL).	N/A	34.73% (n = 91)	N/A	Male.
Singh, S. et al. (2012) [97]	HIV Medicine	retrospective study	European Region	United Kingdom	106	Hospital	N/A	N/A	6.6% (n = 7)	N/A	N/A
Tseng, Z. H. et al. (2012) [52]	Journal of the American College of Cardiology (JACC)	cohort study	Region of the Americas	United States	2,860	Clinic	Stress Score index’. Social support index’.	N/A	N/A	1.54% (n = 44)	Overdoses.
Yaroslavtseva, T. et al. (2012) [98]	European Neuropsychopharmacology	cross-sectional study	European Region	Russia	700	Hospital	Beck Depression Inventory (BDI).	56% (n = 392)	36% (n = 252)	N/A	Drug dependence, alcohol dependence, HIV stigma, depression.
Amiya, R. M. et al. (2013) [169]	Sexually Transmitted Infections	cross-sectional study	South-East Asian region	Nepal	321	Clinic	Beck Hopelessness Scale (BHS). Beck Depression Inventory (BDI).	14% (n = 45)	17% (n = 54)	N/A	Depression, on ART for more than 2 years.
Fernandez-Santos, D. M. Et al. (2013) [53]	Sexually Transmitted Infections	cross-sectional study	Region of the Americas	United States	499	Hospital	N/A	N/A	23.7% (n = 118)	N/A	Smoking, alcohol, psychoactive substances, Intravenous drug usage (IVDU)
Jin, H. et al. (2013) [170]	Journal of Acquired Immune Deficiency Syndromes	cross-sectional study	Western Pacific Region	China	204	Database	N/A	N/A	43.1% (n = 88)	N/A	IDU, depression, low social support, stress, alcohol use disorder.
Joge, U. S. et al. (2013) [171]	Indian Journal of Dermatology, Venereology & Leprology	cross-sectional study	South-East Asian region	India	801	Hospital	N/A	12.25 (n = 98)	N/A	N/A	Male.
Kudryashova H, L. (2013) [54]	Sexually Transmitted Infections	cross-sectional study	Region of the Americas	United States	350	Clinic	N/A	N/A	0.57% (n = 2)	N/A	Substance abuse, mental health.
O’Donnell, J. K. et al. (2013) [55]	American Journal of Epidemiology	cross-sectional study	Region of the Americas	United States	69	Hospital	Hamilton Rating Scale for Depression.	28% (n = 19)	N/A	N/A	Depression, psychiatric comorbidities, marital status, adaptive coping styles stress.
Schadé, A. et al. (2013) [99]	BMC Psychiatry	cohort study	European Region	Netherlands	196	Hospital	Beck Scale for Suicide ideation (BSS).	48% (n = 94)	34% (n = 66)	N/A	Depression, fear, anger, guilt.
Heuvel, L. V. D. et al. (2013) [135]	AIDS Care	cross-sectional study	African region	Zambia	649	Clinic	Mini International Neuropsychiatric Interview (M.I.N.I.).	5.9% (n = 38)	3% (n = 19)	N/A	Depression.
Weber, R. et al. (2013) [100]	HIV Medicine	cohort study	European Region	Switzerland	16,134	Database	N/A	N/A	N/A	6% (n = 28)	N/A
Amiya, R. M. Et al. (2014) [172]	PLoS ONE	cross-sectional study	African region	Nepal	322	Clinic	Beck Depression Inventory (BDI).	14% (n = 45)	N/A	N/A	Depression, lower family support.
Ceccon, R. F. et al. (2014) [16]	Revista de saúde pública	cross-sectional study	South-East Asian region	Brazil	161	Clinic	Suicide (QIS—Suicide Ideation Questionnaire). Suicide Ideation Questionnaire16 adapted by Ferreira & Castela.	50% (n = 80)	N/A	N/A	Age at first sexual intercourse < 15 years, children, poverty, living with HIV for long, violence, female.
Ehren, K. et al. (2014) [136]	Infection	cohort study	European Region	Germany	3,165	Database	N/A	N/A	N/A	4% (n = 7)	N/A
Forbes, K. et al. (2014) [56]	Archives of Disease in Childhood	cross-sectional study	Region of the Americas	United States	54	Palliative care center	N/A	N/A	31.5% (n = 17)	N/A	Sad, hopeless, have had sex against their will, aged 12 or younger at sexual debut, psychosocial support.
Guillemi, S. et al. (2014) [57]	Topics in Antiviral Medicine	cohort study	Region of the Americas	Canada	5,229	Hospital	N/A	N/A	N/A	8.2% (n = 82)	IDU, never having been diagnosed with an AIDS defining illness.
Hogg, R. S. Et al. (2014) [16]	Canadian Journal of Infectious Diseases and Medical Microbiology	retrospective study	Region of the Americas	Canada	5,229	Hospital	N/A	N/A	N/A	1998year: 961 deaths per 100, 000 person-years- 2010year: 2.81 deaths per 100, 000 person years. 1998 year: 25%-2010 year: 1.3%.	Younger age, IDU, higher last CD4 count, never having an AIDS defining illness.
Jovet-Toledo, G. G. et al. (2014) [58]	AIDS Care	Retrospective study	Region of the Americas	Puerto Rico	1,185	Clinic	N/A	N/A	20.4% (n = 242)	N/A	Gender, employment, drug use, sex work.
Kim, et al. (2014) [137]	Journal of the International AIDS Society	cross-sectional study	African region	Malawi	562	Clinic	Beck Depression Inventory-II (BDI-II). Children’s Depression Inventory-II-Short (CDI-II-S).	7.1% (n = 40)	N/A	N/A	Depression.
McManus, H. et al. (2014) [189]	PLoS ONE	cohort study	European Region	Australia	81	Database	N/A	67% (n = 55)	85.18% (n = 69)	N/A	MSM, IDU, unemployment, living alone.
Mollan, K. R. et al. (2014) [59]	Annals of Internal Medicine	cohort study	Region of the Americas	United States	5,332	Hospital	N/A	N/A	8.08% (n = 62)	N/A	Efavirenz.
Passos, S. M. et al. (2014) [17]	AIDS Care	cross-sectional study	Region of the Americas	Brazil	211	Clinic	Brazilian version of Hospital Anxiety and Depression Scale (HAD). Module C of the instrument Mini International Neuropsychiatric Interview 5.0 (MINI).	34.1% (n = 72)	N/A	N/A	Female, age up to 47 years, unemployment, anxiety, depression, abuse or addiction on psychoactive substances.
Anagnostopoulos, A. et al. (2015) [101]	PLoS ONE	cohort study	European Region	Switzerland	4,222	Hospital	N/A	N/A	N/A	0.43% (n = 18)	Female, older age, preserved work ability and higher physical activity, depression, IDU.
Croxford, S. et al. (2015) [102]	HIV Medicine	cohort study	European Region	United Kingdom	83,276	Database	N/A	N/A	N/A	4.25 per 10000 person-years.	N/A
Dabaghzadeh, F. et al. (2015) [185]	Iranian Journal of Psychiatry	cross-sectional study	Eastern Mediterranean region	Iran	150	Clinic	Hospital Anxiety and Depression Scale (HADS). Positive and Negative Suicide Ideation (PANSI).	Suicidal ideation	N/A	N/A	Anxiety, depression, poor physical activity, sleep quality, unemployment, living alone, lack of family support.
Gonzalez-Torres, M. A. et al. (2015) [103]	Neuropsychiatric Disease and Treatment	cross-sectional study	European Region	Spain	25	Hospital	N/A	N/A	72% (n = 18)	N/A	Substance.
Gurm, J. et al. (2015) [60]	CMAJ Open	cohort study	Region of the Americas	Canada	5,229	Database	N/A	N/A	N/A	8.2% (n = 428)	IDU, having no experience with an AIDS-defining illness.
Ogundipe, O. A. et al. (2015) [18]	Archives of Suicide Research	cross-sectional study	African region	Nigeria	295	Hospital	Beck Depression Inventory (BDI).	N/A	13.6% (n = 40)	N/A	Poorer quality of life, unemployment, emotional distress, religion, HIV status non-disclosure, previous suicidal attempt.
Schlebusch, L. et al. (2015) [138]	Depress Research Treatement	cross-sectional study	African region	South Africa	157	Hospital	Beck Hopelessness Scale (BHS). Beck Depression Inventory (BDI).	28.8% (n = 45)	N/A	N/A	Younger age group (age < 30 years), lower level of education, socioeconomic pressures, traditional beliefs.
Wu, Y. L. et al. (2015) [12]	International Journal of STD & AIDS	cross-sectional study	Western Pacific Region	China	184	Hospital	Beck Hopelessness Scale (BHS). Beck Depression Inventory (BDI)..	31% (n = 57)	N/A	N/A	Stigma, depression, anxiety.
Bitew, H. et al. (2016) [139]	Depress Res Treat	cross-sectional study	African region	Ethiopia	393	Hospital	Patients Health Questionnaire version-9 (PHQ 9). Oslo Social Support scale.	33.6% (n = 132)	20.1% (n = 79)	N/A	Female, marital status, depression, CD4 level, opportunistic infection, stigma, poor social support.
Cheung, C. C. et al. (2016) [61]	HIV Medicine	cohort study	Region of the Americas	Canada	8,185	Hospital	N/A	N/A	N/A	0.47 per 100 person-years.	Age, gender, IDU, AIDS diagnoses, CD4 cell counts.
Collins, P. Y. et al. (2016) [140]	Community mental health journal	cross-sectional study	African region	South Africa	594	Clinic	Patients Health Questionnaire version-9 (PHQ 9).	Suicidal ideation	N/A	N/A	Psychological distress, Socioeconomic status.
Croxford, S. et al. (2016) [104]	HIV Medicine	cohort study	European Region	United Kingdom	83,276	Database	N/A	N/A	N/A	0.23% (n = 190)	N/A
de Almeida, S. M. et al. (2016) [62]	Journal of Neurovirology	cross-sectional study	Region of the Americas	Brazil	39	Hospital	Beck Depression Inventory-II (BDI-II).	N/A	18% (n = 7)	N/A	Depression.
Fawzi, M. C. S. et al. (2016) [137]	Pediatrics	cross-sectional study	African region	Rwanda	193	Clinic	N/A	N/A	12% (n = 7)	N/A	Conduct problems, depression.
Kang, C. R. et al. (2016) [173]	AIDS Care	cross-sectional study	Western Pacific region	South Korea	422	Clinic	N/A	44% (n = 193)	11%. (n = 47)	N/A	Young and middle age, living with someone, opportunistic disease, depression, lower social support, psychological status.
Mugisha, J. et al. (2016) [142]	African Health Sciences	cross-sectional study	African region	Uganda	2,400	Clinic	Duong scale (Cronbach’s alpha = 0.81).	12.1%. (n = 290)	6.2% (n = 149)	N/A	Female, depression, post-traumatic stress disorder.
O’Donnell, J. K. et al. (2016) [63]	Journal of Affective Disorders	longitudinal study	Region of the Americas	United States	289	Database	M.I.N.I. neuropsychiatric interview (MINI Plus).	7–19% (n = 21)	N/A	N/A	N/A
Rukundo, G. Z. et al. (2016) [143]	Aids Research and Treatment	cross-sectional study	African region	Uganda	543	Clinic	The Hamilton Rating Scale for Depression (HAM-D). Mini International Neuropsychiatric Interview (MINI).	8.8% (n = 48)	3.1% (n = 17)	N/A	Anger, depression, hopelessness, anxiety, low social support, inability to provide for others, stigma.
Rukundo, G. Z. et al. (2016) [144]	African Journal of AIDS Research	cross-sectional study	African region	Uganda	543	Clinic	Perceived Social Support Questionnaire (PSSQ). HIV Stigma Scale Questionnaire (HSSQ). Beck Hopelessness Scale (BHS).	10% (n = 54)	3%. (n = 17)	N/A	Poor physical health, physical pain, reducing work due to illness, recent HIV diagnosis.
Sherr, L. et al. (2016) [105]	Journal of Virus Eradication	cross-sectional study	European Region	United Kingdom	170	Clinic	N/A	56.6% (n = 96)	N/A	N/A	Female, depression.
Walter, K. N. et al.[64] (2016)	International Journal of STD & AIDS	cross-sectional study	Region of the Americas	United States	170	Hospital	Patients Health Questionnaire version-9 (PHQ 9).	N/A	35.3% (n = 60)	N/A	Poorer emotional, cognitive quality of life.
Alderete, C. et al. (2016) [65]	Salud Mental	cross-sectional study	Region of the Americas	Mexico	115	Hospital	The Hospital Anxiety and Depression Scale. Beck Hopelessness Scale. Plutchik Suicide Risk Scale.	10.4% (n = 12)	N/A	N/A	Depression, Anxiety.
Bantjes, J. et al. (2016) [21]	AIDS Care	cross-sectional study	African Region	South Africa	500	Clinic		24.27% (n = 121)	N/A	N/A	Depression.
Bengtson, A. M. et al. (2017) [66]	Journal of Acquired Immune Deficiency Syndromes	cohort study	Region of the Americas	United States	597	Database	The Addiction Severity Index (ASI)39 assessed medical, drug, alcohol, employment, legal, family/social, and psychiatric problems. The Functional Assessment of Human Immuno-deficiency Virus Infection quality of life instrument (FAHI).	38% (n = 227)	N/A	N/A	Depression, mental health, after ART initiation.
Carrieri, M. P. et al. (2017) [106]	PLOS ONE	cross-sectional study	European Region	France	2,973	Hospital	Patients Health Questionnaire version-9 (PHQ 9).	6.3% (n = 187)	N/A	N/A	Female, men who have sex with men (MSM), discrimination-related social contexts reported, Homelessness, feeling of loneliness.
Croxford, S. et al. (2017) [107]	HIV Medicine	cohort study	European Region	United Kingdom	88,994	Database	N/A	N/A	N/A	2.1 per 10,000 person-years. 1.8% (n = 96)	Male.
Egbe, C. O. et al. (2017) [145]	BMC Public Health	cross-sectional study	African Region	Nigeria	1,187	Clinic	World Mental Health Composite International Diagnostic Interview (WMH-CIDI) questionnaire	2.9% (n = 34)	2.3% (n = 27)	N/A	Alcohol abuse, Depression, marital status, religion.
Ferlatte, O. et al. (2017) [67]	AIDS Care	cross-sectional study	Region of the Americas	Canada	673	Database	World Mental Health Composite International Diagnostic Interview (WMH-CIDI) questionnaire.	22% (n = 150)	5% (n = 33)	N/A	Rejected as a sexual partner, verbally abused, physically abused.
Gebremariam, E. H. et al. (2017) [146]	Psychiatry J	cross-sectional study	African region	Ethiopia	423	Hospital	Diagnostic Interview (CIDI) adopted by World. Mental Health (WMH) Survey Initiative version of the World Health Organization (WHO).	22.5% (n = 95)	13.9% (n = 59)	N/A	Female, not being on HAART, substance, depression, stigma.
Goehringer, F. et al. (2017) [108]	AIDS Research and Human Retroviruses	prospective study	European Region	France	82,000	Clinic	N/A	N/A	N/A	12.5% (n = 10,250)	Socioeconomic difficulty
Kalungi, A. et al. (2017) [143]	BMC Genetics	cross-sectional study	African region	Uganda	600	Clinic	Mental Health (WMH) Survey Initiative version of the World Health Organization (WHO)."	3.3% (n = 20)	N/A	N/A	N/A
Kinyanda, E. et al. (2017) [148]	Journal of Affective Disorders	cross-sectional study	African region	Uganda	899	Clinic	Major Depression disorder (MDD) as defined by DSM IV in the MINI Plus module. Moderate to high risk for suicidality’ (MHS) as defined in the suicidality module of the MINI Plus to be a score of nine.	2.78% (n = 25)	N/A	N/A	Socio-demographic, vulnerability/protective, stress, impaired psychosocial functioning.
Lemsalu, L et al. (2017) [109]	AIDS and Behavior	cross-sectional study	African region	Estonia	828	Hospital	Composite International Diagnostic Interview (CIDI-10.0). WHO Quality of Life (WHOQOL-HIV-BREF).	36% (n = 288)	20% (n = 160)	N/A	Younger age, incarceration, abused alcohol, IDU, having lived with HIV for more than 10 years, depressed.
Liu, Y. et al. (2017) [174]	AIDS Care	cross-sectional study	Western Pacific Region	China	557	Hospital	N/A	25% (n = 139)	N/A	N/A	After HIV diagnosis, HIV-related clinical symptoms, stress, Depression, anxiety, social support.
Oladeji, B. D. et al. (2017) [149]	Journal of the International Association of Providers of AIDS Care	cross-sectional study	African region	Nigeria	828	Clinic	WHO Quality of Life (WHOQOL-HIV-BREF).	15.1% (n = 125)	3.9% (n = 32)	N/A	Female, Depression, anxiety.
Paparizos, V. et al. (2017) [110]	InfezMed	cross-sectional study	European Region	Greece	1,884	Hospital	N/A	N/A	N/A	1.48% (n = 28)	Psychiatric co-morbidity, depression, bipolar disorder, anxiety disorder.
Quinlivan, E. B. et al. (2017) [68]	AIDS and Behavior	Retrospective study	Region of the Americas	United States	4,099	Hospital	Patients Health Questionnaire version-9 (PHQ 9).	8.6% (n = 352)	N/A	N/A	3 years since HIV diagnosis, HIV RNA >50 copies/ml.
Rodriguez, V. J. et al. (2017) [13]	AIDS Care	cross-sectional study	African Region	South Africa	673	Clinic	The Edinburgh Postnatal Depression Scale (EPDS-10, α = 0.75).	39% (n = 262)	N/A	N/A	Female, partner violence, stigma.
Wang, H. et al. (2017) [175]	Journal of Central South University. Medical sciences	cross-sectional study	Western Pacific Region	China	504	Clinic	Self-rating Depression Scale. Beck Scale for Suicide Ideation-Chinese Version.	27.2% (n = 137)	N/A	N/A	Male, gay, suicide history, anxiety, depression.
Wong, M. et al. (2017) [150]	Archives of Women’s Mental Health	cohort study	African Region	South Africa	625	Clinic	The Edinburgh Postnatal Depression Scale (EPDS)	N/A	6% (n = 36)	N/A	Age, depression.
Woollett, N. et al. (2017) [151]	Journal of Child & Adolescent Mental Health	cross-sectional study	African Region	South Africa	343	Hospital	The MINI International Psychiatric Interview for children and adolescents suicide scale. Child Depression Inventory Short Form, which has a high correlation.	N/A	24% (n = 82)	N/A	Depression, anxiety, PTSD.
Ashaba, S. et al. (2018) [152]	Global Mental Health	cross-sectional study	African Region	Uganda	224	Hospital	Six-item Internalized AIDS-Related Stigma Scale. Mini International Neuropsychiatric Interview for Children and Adolescents (MINI-KID).	N/A	13% (n = 29)	N/A	Depression, stigma, bullying.
Brennan, C. et al. (2018) [69]	Innovations in Clinical Neuroscience	cross-sectional study	Region of the Americas	Columbia	1,056	Clinic	The electronic Columbia-Suicidality Severity Rating Scale (eC-SSRS).	14% (n = 148)	9% (n = 96)	N/A	ARTs, depression.
Chang, J. L. et al. (2018) [153]	Annals of Internal Medicine	cohort study	African Region	Uganda	694	Clinic	N/A	6.2% (n = 19)	N/A	N/A	Depression.
Hentzien, M., A. et al. (2018) [111]	HIV Medicine	cross-sectional study	European Region	France	349	Database	N/A	N/A	N/A	4.1% (n = 99)	Not having children, psychological morbidity, substituted drug consumption, alcohol intake > 20 g/day, abuse, Depression, psychotropic drugs.
Hsing-Fei, L. U. et al. (2018) [176]	Journal of Nursing	cross-sectional study	Western Pacific Region	Taiwan	114	Hospital	Beck Scale for Suicidal Ideation (BSS). Beck Depression Index 2nd version (BDI-II). Meaning in Life Questionnaire (MLQ).	27.2% (n = 31)	14% (n = 16)	N/A	Suicide ideation: duration since being diagnosed HIV-positive, level of education, depression. Suicide attempt: depression.
Loeliger, K. B. et al. (2018) [17]	The Lancet. HIV	case-control study	Region of the Americas	United States	1,350	Hospital	N/A	N/A	N/A	7.6% (n = 13)	Drug overdose, age (>/ = 50 years, lower CD4 count (<200 cells per mu), high number of comorbidities, virologic failure), unmonitored viral load.
Loeliger, K. B. et al. (2018) [70]	Topics in Antiviral Medicine	retrospective study	Region of the Americas	United States	1,350	Prisons	N/A	N/A	N/A	8% (n = 13)	Age> = 50 years, lower CD4 count, high number of comorbidities, virologic failure, unmonitored viral load.
Lopez, J. D. et al. (2018) [71]	Aids and Behavior	cross-sectional study	Region of the Americas	United States	648	Hospital	The Patient Health Questionnaire-9 (PHQ-9).	13% (n = 81)	N/A	N/A	Anxiety, have unsuppressed viral loads, consider themselves to be homeless.
Lu, H. F. et al. (2018) [177]	Hu Li Za Zhi	cross-sectional study	Western Pacific Region	Taiwan	114	Prisons	Beck Scale for Suicidal Ideation (BSS,Cronbach’s α.89). Beck Depression Index 2nd version (BDI-II, Cronbach’s α.89). Meaning in Life Questionnaire (MLQ). The Multidimensional Scale of Perceived Social Support Chinese version, (MSPSS-C, Cronbach’s α = .96)	27.2% (n = 31)	14% (n = 16)	N/A	Education level, depression.
Malava, J. K. et al. (2018) [112]	Malawi Medical Journal	cross-sectional study	European Region	United Kingdom	206	Hospital	Patients Health Questionnaire version-9 (PHQ 9).	16% (n = 33)	N/A	N/A	Alcohol use, depression.
Pinto, A. N. et al. (2018) [196]	AIDS Research and Human Retroviruses	cross-sectional study	Western Pacific Region	Australia	138	Clinic	N/A	N/A	N/A	4% (n = 5)	N/A
Rodriguez, V. J. et al. (2018) [13]	AIDS and Behavior	cohort study	African region	South Africa	681	Database	N/A	39% (n = 265)	N/A	N/A	Prenatal, intimate partner violence, depression, increased income, stigma, younger age, disclosure of HIV status to partner.
Rodriguez, V. J. et al. (2018) [72]	AIDS Care	cross-sectional study	Region of the Americas	Argentina	118	Clinic	N/A	35.6% (n = 42)	N/A	N/A	Female, younger, unemployed, stigma.
Sherr, L. et al. (2018) [154]	AIDS Care—Psychological and Socio-Medical Aspects of AIDS/HIV	cross-sectional study	African region	South Africa	1,058	Clinic	The Child Depression Inventory short form. Anxiety was measured using the Children’s Manifest Anxiety Scale. Posttraumatic stress symptoms. Suicidality/self-harm was measured using the Mini International Neuropsychiatric Interview.	4.1% (n = 42)	N/A	N/A	Substance use, depression, anxiety.
Sumari-de Boer, M. Et al. (2018) [155]	Tropical Medicine and International Health	cross-sectional study	African region	Tanzania	365	Hospital	The Hospital Anxiety and Depression Scale (HADS). The Mini-International Neuropsychiatric Interview (MINI).	10% (n = 36)	N/A	N/A	Alcohol use, depression.
Tang, X. et al. (2018) [12]	Neuropsychiatric Disease and Treatment	cross-sectional study	Western Pacific Region	China	504	Clinic	Self-Rating Depression Scale (SDS). Social support rate scale (SSRS, Cronbach’s α = 0.762).	27.2% (n = 137)	N/A	N/A	Depression, social support, emotion-focused coping and problem-focused coping.
Wang, W. et al. (2018) [9]	PLOS ONE	cross-sectional study	Western Pacific Region	China	465	Hospital	Center for Epidemiological Studies Depression (CES-D, Cronbach’s alpha = 0.914).	31.6% (n = 147)	N/A	N/A	Low social support, depression, stigma, older age, low education level, married, having children, psychosocial.
Zarei, N. et al. (2018) [186]	AIDS Research and Treatment	cross-sectional study	Eastern Mediterranean region	Iran	351	Hospital	To evaluate stigma and discrimination, 12 items (four-choice question, 7 for internal and 5 for external/social stigma).	15.4% (n = 54)	N/A	N/A	Quality of life, spiritual beliefs, stigma, age, gender, marital status.
Zeng, C. et al. (2018) [178]	BMC Public Health	cross-sectional study	Western Pacific Region	China	411	Clinic	The Center for Epidemiologic Studies Depression Scale (CES-D), a 20-item scale with four dimensions, including depressed affect, positive affect, somatic and retarded activity, and interpersonal problems. Internal consistency estimate of reliability of the scale was good (Cronbach’s alpha = 0.93).	32.4% (n = 133)	9% (n = 37)	N/A	Depression.
Casale, M. et al. (2019) [156]	Journal of Affective Disorders	cross-sectional study	African region	South Africa	1,053	Hospital	Mini International Psychiatric Interview for Children and Adolescents Suicidality and Self-harm subscale. Children’s Depression Inventory (CDI 10-item short form) α = 0.64.	6.2% (n = 66)	N/A	N/A	Depression, stigma.
Croxford, S. et al. (2019) [113]	HIV Medicine	retrospective study	European Region	United Kingdom	166	Database	N/A	N/A	N/A	7% (n = 12)	N/A
Knieps, L. et al. (2019) [114]	HIV Medicine	cohort study	European Region	Germany	81	Database	N/A	N/A	N/A	6.2% (n = 5)	N/A
Kreniske, P. et al. (2019) [17]	Journal of Adolescent Health	cross-sectional study	Region of the Americas	United States	340	Clinic	N/A	N/A	NA	N/A	Age 14–18.
Li, Y. et al. (2019) [179]	Journal of Medical Internet Research	longitudinal study	Western Pacific Region	Taiwan	300	Hospital	The Chinese version of the CES-D scale. Perceived Stress Scale (PSS-10).	45% (n = 135)	9.7% (n = 29)	N/A	Stress, depression.
Lu, H. F. et al. (2019) [180]	Journal of advanced nursing	longitudinal study	Western Pacific Region	Taiwan	113	Hospital	Beck Depression, Inventory-II (BDI-II). Meaning in Life Questionnaire and the Multidimensional Scale of Perceived Social Support at baseline.	27.2% (n = 31)	14.7% (n = 17)	N/A	Education level, social support from family, depression.
Mandell, L. N. Et al. (2019) [73]	AIDS and behavior	cross-sectional study	Region of the Americas	Argentina	360	Hospital	N/A	21% (n = 76)	N/A	N/A	Younger age, depression.
Peltekis, A. et al. (2019) [115]	Psychiatrike = Psychiatriki	cross-sectional study	European Region	Greece	191	Clinic	Patients Health Questionnaire version-9 (PHQ 9).	9.2% (n = 17)	14% (n = 27)	N/A	N/A
Ruffieux, Y. et al. (2019) [116]	Journal of the International AIDS Society	longitudinal study	European Region	Switzerland	20,136	Hospital	N/A	N/A	N/A	1% (n = 204)	Gender, male, nationality, centers for disease control and prevention clinical stage, IDU, mental health, psychiatric treatment.
Sarna, A. et al. (2019) [181]	Archives of Women’s Mental Health	retrospective study	South-East Asian region	India	200	Clinic	N/A	N/A	23% (n = 46)	N/A	Depression.
Shim, E. J. et al. (2019) [181]	International journal of behavioral medicine	cohort study	Western Pacific Region	South Korea	202	Database	The Hospital Anxiety and Depression Scale. Mini-International Neuropsychiatric Interview suicidality module.	20% (n = 40)	N/A	N/A	Thwarted belongingness (TB), perceived burdensomeness (PB), depression.
Tyree, G. A. et al. (2019) [74]	Journal of Affective Disorders	cross-sectional study	Region of the Americas	United States	1,002	Clinic	Patient Health Questionnaire 9 (PHQ-9)	38.4% (n = 385)	N/A	N/A	Depression.
Wang, Y. Y. et al. (2019) [182]	Psychology, health & medicine	cross-sectional study	Western Pacific Region	China	410	Clinic	Generalized Anxiety Disorder score (GAD-7)	10.7% (n = 44)	3.2% (n = 13)	N/A	Unemployment, age, CD4 lymphocyte counts, anxiety.
Wonde, M. et al. (2019) [75]	PLoS ONE	cross-sectional study	Region of the Americas	United States	413	Hospital	Patient Health Questionnaire 9 (PHQ-9)	27.1% (n = 112)	16.9% (n = 70)	N/A	Suicide ideation: Female, family death, WHO clinical stage III of HIV, WHO clinical stage IV of HIV, depression, perceived HIV stigma. Suicide attempt: female, opportunistic infections, WHO clinical stage III of HIV, depression, poor social support.
Adeyemo, S. et al. (2020) [157]	Child and Adolescent Psychiatry and Mental Health	cross-sectional study	African region	Nigeria	201	Hospital	Mini International Neuropsychiatric Interview for children and adolescents (MINI-Kid).	35.3% (n = 71)	N/A	N/A	High ACE score, physical abuse, emotional abuse, depression.
Bi, F. Y. et al. (2020) [183]	Psychology Health & Medicine	cross-sectional study	Western Pacific Region	China	557	Clinic	Social Support Rating Scale (SSRS). Chinese HIV/AIDS Stress Scale (CSS-HIV). Patient Health Questionnaire (PHQ-9).	24.95% (n = 139)	N/A	N/A	Low social support, depression.
Durham, M. D. et al. (2020) [76]	Preventive Medicine	prospective study	Region of the Americas	United States	6,706	Hospital	N/A	3.3% (n = 224)	N/A	N/A	<50 years old, non-Hispanic/Latino black, have CD4+ cell count <350 cells/mm3, have a viral load ≥50 copies/mL, have stopped antiretroviral therapy, alcohol dependence, drug overdose.
Fontela, C. et al. (2020) [117]	Scientific reports	Cohort study	European Region	Spain	1,059	Database	N/A	N/A	N/A	1.32% (n = 14)	N/A
Kindaya, G. G. et al. (2020) [13]	HIV/AIDS—Research and Palliative Care	cross-sectional study	African region	Ethiopia	412	Hospital	N/A	24.3% (n = 100)	12.6% (n = 52)	N/A	Suicide ideation: extreme poverty, living alone, widowed, CD4 level less than 250, current alcohol use. Suicidal attempt: urban residence, stage IV HIV, family history of suicide, depression.
Knettel, Brandon A. et al. (2020) [158]	AIDS	cross-sectional study	African region	Tanzania	200	Clinic	N/A	12.8% (n = 26)	N/A	N/A	Anxiety, HIV stigma.
Mebrahtu, H. et al. (2020) [159]	AIDS & Behavior	cross-sectional study	African region	Zimbabwe	562	Hospital	Parental stress index-short form (PSI-SF). Common mental disorders (CMDs).	30.43% (n = 171)	N/A	N/A	Younger, unmarried, experienced moderate to severe hunger, had elevated parental stress, depression symptoms.
Nishijima, T. et al. (2020) [184]	AIDS	cohort study	Western Pacific Region	Japan	277	Clinic	N/A	N/A	N/A	7.76% (n = 14)	
Ophinni, Y. et al. (2020) [10]	BMC Psychiatry	cross-sectional study	South-East Asian region	Indonesia	86	Clinic	Symptom Checklist-90 (SCL-90).	23.3% (n = 20)	N/A	N/A	Depression, anxiety, non-marital status, CD4 count < 500 cells/μl, efavirenz use.
Sereda, Y. et al. (2020) [118]	Journal of the International AIDS Society	cross-sectional study	European Region	Ukraine	191	Hospital	Primary outcomes were HIV stigma (Berger scale). Substance use stigma (Substance Abuse Stigma Scale).	N/A	28% (n = 54)	N/A	HIV stigma.
Gizachew et al. (2021) [196]	Annals of General Psychiatry	cross-sectional study	African region	Ethiopia	326	Hospital	Composite International Diagnostic Interview (CIDI). Patient Health Questionnaire for Anxiety and Depression (PHQ-4)	16% (n = 52)	7.1% (n = 23)	N/A	Low monthly income, living alone, suicidal thought before knowing seropositive status, family history of suicide, experiencing mild and moderate-to-severe depression and anxiety symptoms, use of khat.
Tamirat et al. (2021) [197]	HIV/AIDS	cross-sectional study	African region	Ethiopia	395	Hospital	Composite International Diagnostic Interview (CIDI). Patient Health Questionnaire (PHQ-9).	9.40% (n = 37)	3.3% (n = 13)	N/A	Low body mass index, stages three and above illnesses, depression, poor social support, and fair and poor adherence.
Wang et al. (2021) [199]	Journal of Affective Disorders	cross-sectional study	Western Pacific Region	China	494	Hospital	Personal Social Capital Scale (PSCS-8).	32.59% (n = 161)	12.15% (n = 60)	N/A	Social capital.
Zewdu et al. (2021) [198]	BMC Pregnancy and Childbirth	cross-sectional study	African region	Ethiopia	414	HIV clinics	Composite International Diagnostic Interview (CIDI).	8.20% (n = 34)	N/A	N/A	Perinatal depression, not disclosed HIV status, unplanned pregnancy.

### Qualitative synthesis

Qualitative synthesis was conducted using data extraction findings to explore the key themes within the selected studies. Three researchers independently conducted the qualitative synthesis on the baseline risk factors of the participants and suicidal ideation rate, suicide attempt rate, and completed suicide rate (Table 1). We created a structural model related to the consistent risk factors after final agreement among all authors.

## Results

### Study identification

**Fig 1:**
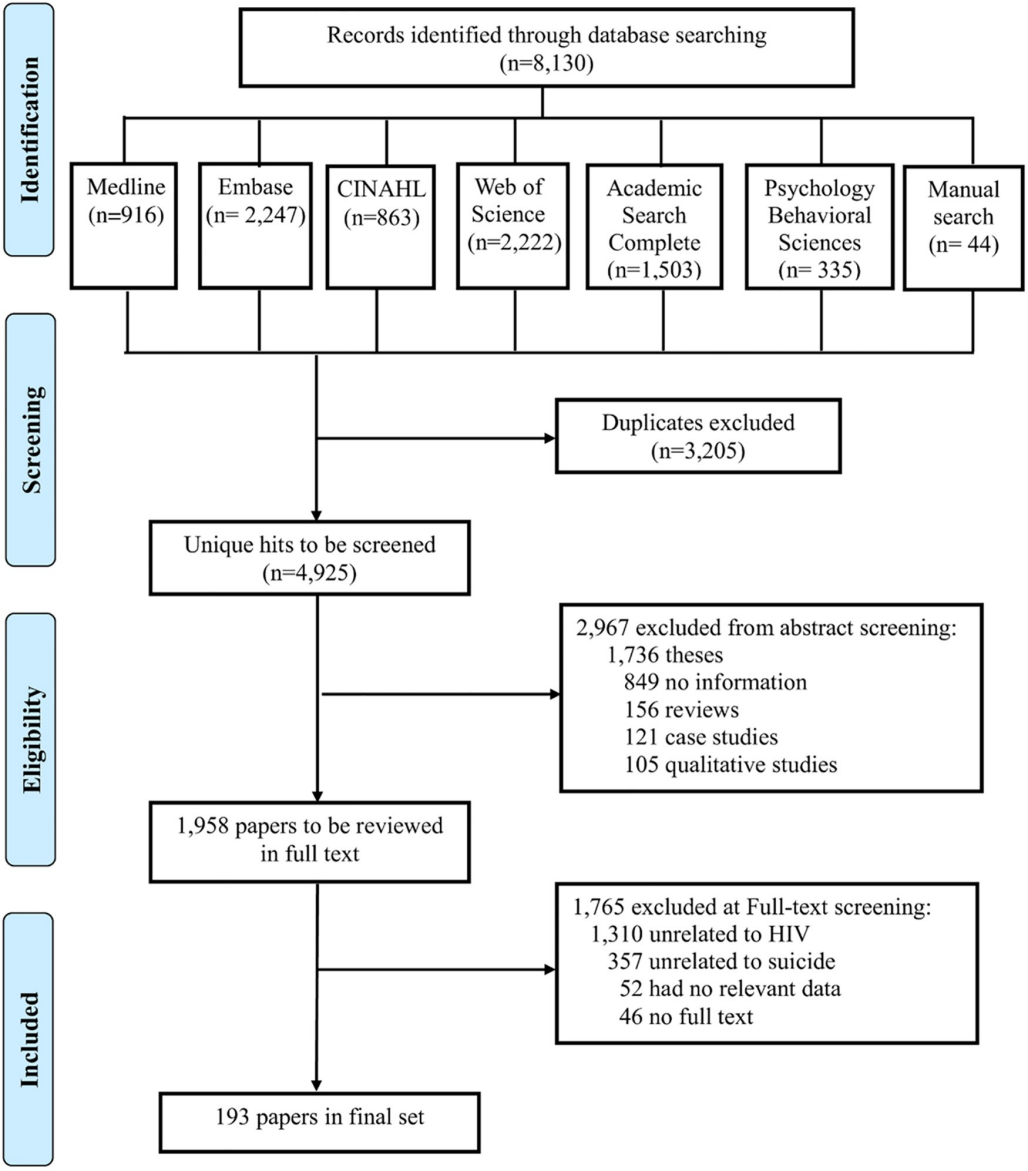
PRISMA style flow diagram included studies.

After searching the six databases, 8,055 articles published from January 1, 1988, to July 8, 2021, were identified (Ovid Medline, 878; Embase, 2,123; CINAHL, 815; Web of Science, 2,450; Academic Search Complete, 1,357; Psychology & Behavioral Sciences Collection, 388; manual search, 44). After removing 3,205 duplicate articles, 4,850 articles were screened for the title and abstract. Of these, 1,954 met the inclusion criteria and they were eligible to be considered for reading in the systemic review. The remaining 2,896 articles were excluded for the following reasons: 1,716 did not mention HIV/AIDS; 820 did not mention suicidal behavior; 262 did not clearly assess the outcome variables; and 98 were not available in full text format. After quality assessment, 193 articles were included in this scoping review (Fig 1). All included studies were published as a full article in peer-reviewed journal.

### Characteristics of the included studies

**Fig 2:**
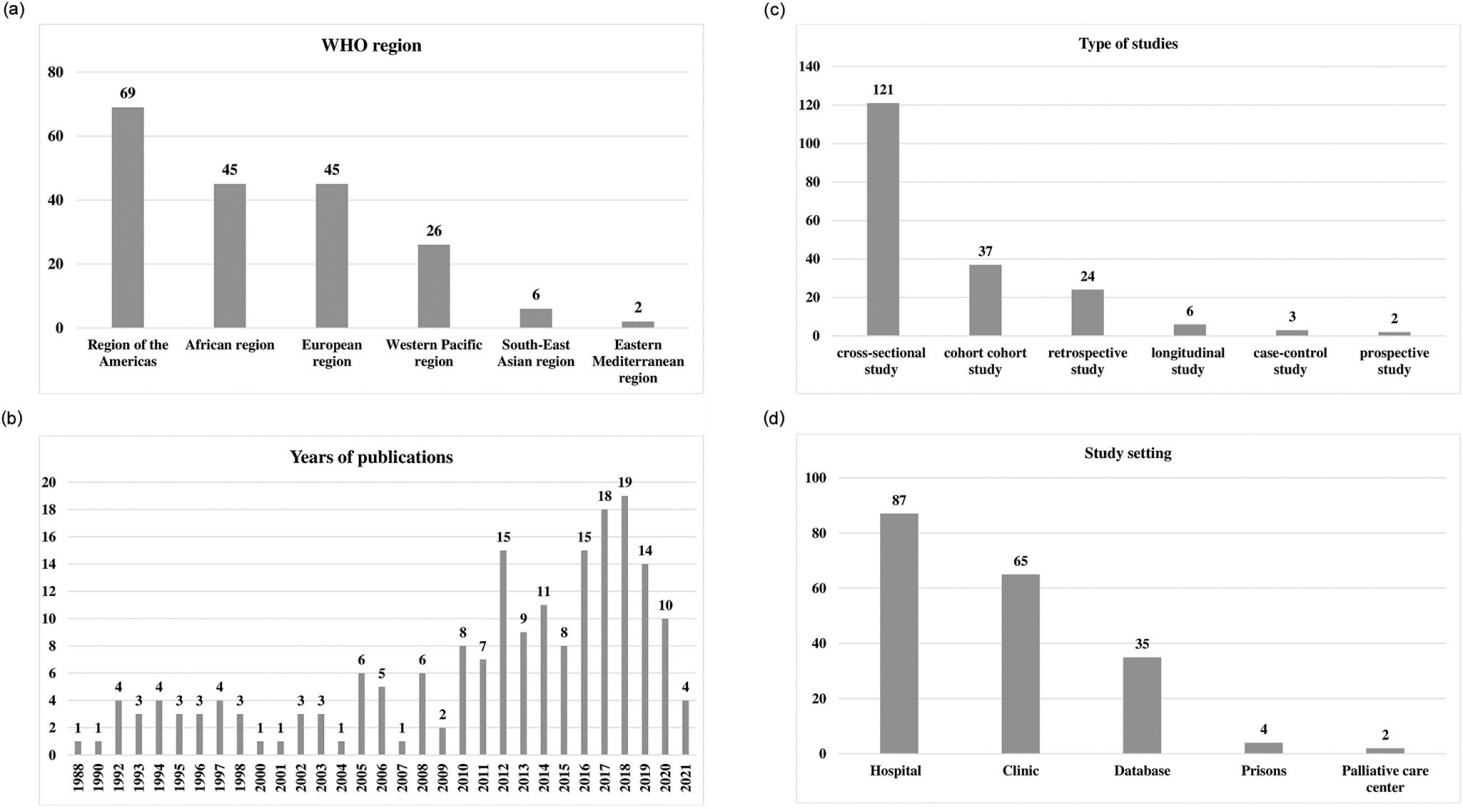
A. Studies distribution according to world health organization (WHO) regions. B. Years of publications. C. Types of studies. D. Study setting.

According to the WHO regions [22], 69 studies were performed Region of the Americas [9, 17, 20, 24–80], followed by 45 in Europe Region [5, 29, 31, 77–121], 45 in Africa Region [13, 18, 21, 126–164], 26 in Western Pacific Region [9, 165–190], 2 in the Eastern Mediterranean Region [191, 192], and 6 South-East Asian Region[9, 16, 168, 169, 171, 181] (Fig 2A). A total of 130 articles were published during the past 10 years (2011–2021) (Fig 2B).

There were 121 studies conducted with cross-sectional study designs, 37 cohort studies, 24 retrospective studies, six longitudinal studies, three case-control studies, and two prospective studies (Fig 2C). According to the study settings, 88 studies were conducted in a hospital setting, 35 referenced databases, 64 were conducted in clinics or community centers, 4 were performed in prisons, and 2 were done at palliative care centers (Fig 2D).

107 articles used measurement tools to identify the condition of suicidal behavior and risk factors. There were 66 articles on suicidal ideation, 38 on suicidal attempts, 47 on completed suicide, 39 on suicidal ideation and attempts, 1 on suicidal ideation and death by suicide [95], and three on suicidal ideation, suicide attempts, and completed suicides [78, 79, 91]. Most of the studies evaluated risk factors; however, 23 did not mention risk factors (Table 1).

### Epidemiology of suicidal behaviors among PLHIV

#### Suicidal ideation around the world

The Pre HAART era from 1990 to 1996, suicidal ideation prevalence rate is 28.6 to 55% in United States [24, 27, 187, 188]; 50.5% in United Kingdom [79]; and 1.18% in Spain [78].

In the Post-HAART era, from 1997 to 2021, the suicidal ideation prevalence rate was 60% to3.3% in the United States [10, 20, 34–36, 39, 41, 43, 44, 45–49, 55, 63, 66, 68, 71, 74–76, 133]; 69% to 16% in the United Kingdom [80, 105, 112]; 13.2% to 6.3% in France [82, 85, 95, 106]; 9.2% in Greece [115]; 11.4% to 6.2% in Africa [13, 21, 125, 126, 130, 134, 135, 138, 154, 156]; 8.8% to 6.2% in Uganda [142–144, 147, 148, 153]; 34.7% to 35.3% in Nigeria [129, 145, 149, 157]; 35.6% to 21% in Argentina [75, 77]; 67% in Australia[195]; 50% in Brazil [16, 17]; 9.2% to 22% in Canada [50, 67]; 14% in Columbia [69]; 36% in Estonia [109]; 33.6% to 8.2% in Ethiopia [13, 139, 146, 190–192]; 14% in Nepal [174, 177]; 48% in the Netherlands [99]; 34% to 32.6% in China [9, 12, 164, 167, 174, 175, 178, 182, 183, 193]; 39.3% to 27.2% in Taiwan [13, 161, 176, 177, 179, 180]; 10% to 12.8% in Tanzania [155, 158]; to 23.3% in Indonesia [9]; 10.4% in Mexico [65]; 7.1% in Malawi [137]; 12.1% in Romania [92]; 56% in Russia [98]; 44% to 20% in Korea [173, 181]; 15.4% in Iran [186]; and 30.4% in Zimbabwe [159] (S1 Table).

#### Suicide attempts around the globe

In the Pre-HAART era, from 1994 to 1996, the suicidal attempt prevalence rate was 21.8% to 42.8% in the United States [28, 160]; 21.4% in the United Kingdom [79]; and 4.02% in Spain [78].

In the Post-HAART era, from 1997 to 2021, the suicidal attempt prevalence rate was 21% to 16.9% in the United States [20, 37, 38, 43–45, 45, 53, 54, 56, 59, 64, 75]; 31% to 6.6% in the United Kingdom [80, 89, 91, 93, 96, 97]; 22% in France [120, 121]; 14% in Greece [115]; 72% in Spain [103]; 54% to 6% in Africa [123, 127, 135, 150, 151]; 17.4% to 13% in Uganda [124, 128, 132, 142–144, 152]; 9.3% to 2.3% in Nigeria [18, 129, 145, 149]; 27% to 85.2% in Australia [189, 194]; 18% in Brazil [62]; 10.3% to 5% in Canada [50, 67]; 9% in Columbia [69]; 20% in Estonia [109]; 17% in Nepal [169]; 34% in the Netherlands [99]; 17% in the Nepal [169]; 8% to 12.2% in China [164, 178, 179, 182, 193]; 26.7% to 9.7% in Taiwan [162, 165, 166, 176, 177, 179, 180]; 23% in India [181]; 36% in Russia [98]; 11% in Korea, 28% in Ukraine [118]; and 20.4% in Puerto Rico [58] (S2 Table).

#### Distribution of death due to suicide around the world

In the Pre-HAART era, from 1988 to 1996, the completed suicide incidence rate was 680.56–4.9per 100,000 person years in the United States [23, 25, 30, 32, 195]; 0.5% in the United Kingdom [79]; 25% in the Sweden [77]; 0.47% in the Spain [78]; and 8.13% to 13% in the Netherlands [29, 31]. The death rate due to suicide went from 0.68 per 100 person-years in 1988 to 0.05 in 1996 in the United States [23, 25, 30, 32].

In the Post-HAART era, from 1997 to 2020, the completed suicide prevalence rate was 8.7% to 7.6% in the United States [17, 33, 47, 52, 70, 84, 86, 87]; 12.9% to 7% in the United Kingdom [88, 91, 102, 104, 107, 113]; 6% to 4.1% in France [81, 83, 95, 108, 111]; 1.48% in Greece [110]; 0.6%–1.3% in Spain [90, 117]; 4% to 6.2% in Germany [114, 136]; 6% to 1% [104, 105, 120]; 4% in Australia [196]; 7% to 8.2% in Canada [16, 61]; 7.8% in Japan [184]; 5.5% in Taiwan [163]; 15.5% in Thailand [168]; and 38.6% in Denmark [94]. The death due to suicide incidence rate went from 0.03 per 100 person years in 2010 to 0.47 in 2016 in Canada [16, 61], 0.16 per 100 person-years in Switzerland [5], and was 0.04 per 100 person years in 2015 to 0.02 in 2017 in UK [102, 107] (S3 Table).

### Risk factors for suicide behavior among PLHIV

**Fig 3:**
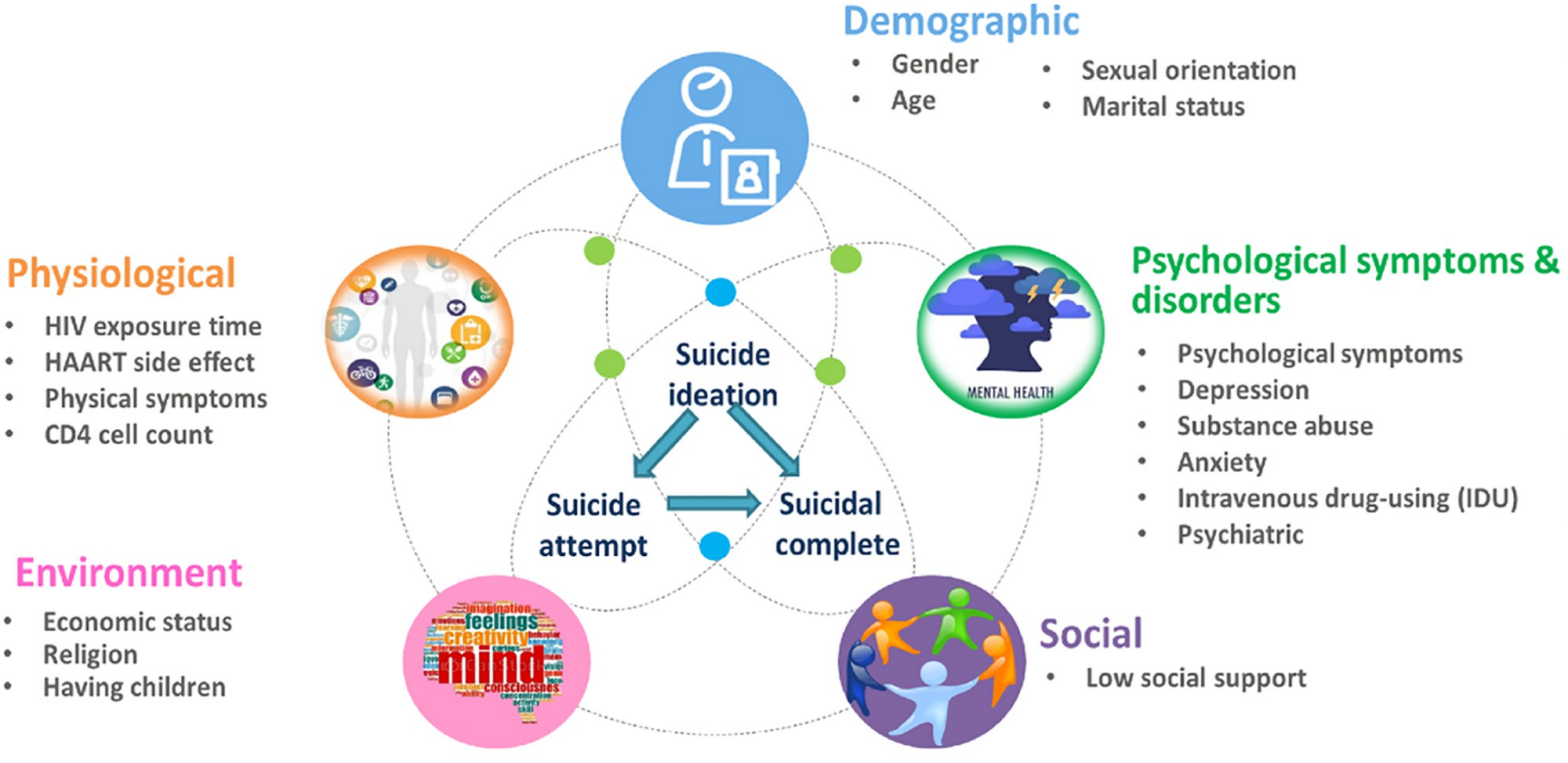
Relationship of suicide type and risk factor among people living HIV as a model there are 5 variations in people living with hiv suicide model. The factors are from risk factors in data which study suicide ideation, suicide attempt and completed suicide.

In this review, the identified causes of death by suicide included drug overdose, gunshot, jumping, drug poisoning, suffocation, and cutting wrists (Table 2). Additionally, we found that suicide-related risk factors included demographic, physiological, social, environmental, and psychological factors (Fig 3).

**Table 2:** Risk factors of suicide among people living with HIV.

Suicide type Risk factors		Suicidal ideation	Suicide attempt	Suicidal complete
Demographic	Gender (♂&♀) inconsistent	Gender: 1 article [192]	Gender:1 article [61]	Gender: 1 articles [64]
		Male:6 articles [9, 40, 49, 131, 176, 180]	Male: 3 articles [38, 100, 125]	Male: 7 articles [5, 33, 34, 81, 90, 111, 120]
		Female:15 articles [14, 16, 18, 45, 75, 79, 84, 109, 110, 133, 137, 143, 146, 150, 153]	Female: 13 articles [15, 43, 45, 79, 84, 124, 132, 133, 136, 143, 146, 150, 153]	Female: 3 articles [41, 92, 105]
	Age (Young, middle, and old age) inconsistent	Age (all ages):2 article [188, 192]	Age (all ages): 1 article [188]	Age (all ages): 2 articles [26, 64]
		Young: 7 articles [13, 16, 75, 77, 113, 138, 164]	Young: 2 articles [58, 113]	Young: 1 article [60]
		Middle: 5 articles [10, 18, 80, 142, 178]	Middle: 3 articles [43, 124, 178]	Middle: 4 articles [5, 24, 34, 73]
		Older:1 article [9]	Older: 0 article	Older: 2 articles [17, 105]
	Sexual orientation inconsistent	Sexual orientation: 2 articles [37, 70]	Sexual orientation: 3 articles [58, 61, 70]	Sexual orientation: 1 article [81]
		Homosexual: 4 articles [40, 110, 180, 195]	Homosexual: 1 article [195]	Homosexual: 0 article
		Heterosexual:1 article [49]	Heterosexual: 0 article	Heterosexual: 0 article
	Low education level	5 articles [9, 142, 181, 181, 185]	4 articles [124, 181, 181, 185]	0 articles
	Marital status inconsistent	Single: 2 articles [11, 164]	Single: 0 article	Single: 1 article [175]
		Married: 4 articles [57, 143, 149, 192]	Married: 3 articles [107, 143, 149]	Married: 1 article [63]
		Widow: 1 article [162]	Widow: 1 article [162]	Widow: 0 article
Psychological symptoms & disorders	Stigma	14 articles [9, 12, 13, 14, 42, 75, 102, 143, 148, 150, 160, 163, 188, 192]	6 articles [79, 102, 143, 148, 150, 156]	0 article
	Psychological symptoms consistent	9 articles [9, 37, 49, 115, 144, 171, 178, 179, 194]	3 article [56, 171, 178]	1 article [115]
	Stress	7 articles [42, 57, 152, 164, 169, 179, 184]	5 articles [125, 132, 169, 175, 184]	0 article
	Hopelessness	3 articles [110, 148, 194]	2 articles [58, 148]	0 article
	Anger	2 articles [103, 148]	2 articles [103, 148]	0 article
	Perceived burdensomeness (PB)	2 articles [35, 132, 187]	0 article	0 article
	Fear	1 article [103]	1 article [103]	0 article
	Guilt	1 article [103]	1 article [103]	0 article
	Depression consistent	59 articles [9–11, 18, 21, 22, 25, 28, 39, 42, 45, 48, 50, 57, 66, 68, 69, 72, 75, 77–79, 89, 99, 102, 103, 109, 113, 116, 119, 129, 130, 134, 135, 137, 139, 141, 143, 146, 147, 149, 150, 152, 153, 157–164, 169, 170, 172, 174, 176–185, 187, 189, 193, 194]	36 articles [21, 43, 45, 65, 72, 79, 82, 93, 102, 103, 113, 125, 128, 136, 139, 143, 145, 146, 148–150, 153, 156, 162, 169–171, 175, 178, 181, 181, 183–186, 200]	5 articles [41, 105, 114, 115, 173]
	Substance abuse consistent	Substance abuse: 10 articles [9, 18, 21, 28, 52, 96, 102, 150, 158, 159]	Substance abuse: 9 articles [21, 39, 52, 55, 56, 102, 107, 125, 150]	Substance abuse: 1 article [115]
		Drug abuse: 1 article [48]	Drug abuse: 2 articles [101, 102]	Drug abuse: 0 article
		Alcohol abuse: 5 articles [80, 113, 116, 149, 162]	Alcohol abuse: 5 articles [101, 102, 113, 132, 149, 154, 162, 175]	Alcohol abuse: 1 article [88]
	Anxiety consistent	16 articles [11, 12, 18, 28, 45, 68, 74, 148, 153, 158, 163, 169, 170, 179, 180, 188]	9 articles [45, 93, 119, 125, 139, 148, 153, 155, 169–171, 188]	1 article [114]
	Intravenous drug-using (IDU) consistent	3 articles [82, 113, 195]	6 articles [55, 82, 113, 125, 175, 195]	11 articles [5, 30, 41, 59, 60, 63, 64, 82, 94, 105, 120]
	Post-traumatic stress disorder (PTSD)	1 article [146]	2 articles [127, 146]	0 article
	Psychiatric consistent	4 articles [36, 57, 69, 96]	4 articles [38, 132, 136, 170]	3 articles [98, 114, 120]
	Major mood disorder	3 articles [21, 28, 182]	2 articles [19, 21]	1 article [120]
Physiological	HIV exposure time consistent	HIV exposure time: 1 article [49]	HIV exposure time: 0 article	HIV exposure time: 1 article [201]
		Early time: 4 articles [71, 83, 147, 179]	Early time: 3 articles [83, 125, 147]	Early time: 4 articles [33, 83, 88, 98]
		Long time: 4 articles [16, 79, 113, 162]	Long time: 3 articles [79, 113, 162]	Long time: 1 article [5, 111]
	HAART side effect consistent	ART (efavirenz): 4 articles [11, 69, 72, 174]	ART (efavirenz): 3 articles [62, 72, 174]	ART (efavirenz): 0 article
		HAART side effect: 3 articles [49, 80, 151]	HAART side effect: 0 article	HAART side effect: 1 article [166]
		Not side effect: 1 article [150]	Not side effect: 1 article [150]	Not side effect: 0 article
	Physical symptoms consistent	4 articles [37, 49, 147, 152]	2 articles [147, 171]	1 article [73]
	CD4 cell count consistent	5 articles [11, 80, 96, 143, 162, 188, 195]	3 articles [162, 165, 188]	5 articles [17, 60, 64, 73, 90]
	Unmonitored viral load	2 articles [74, 80]	0 article	2 articles [17, 73]
	Opportunistic infection	3 articles [79, 143, 178]	3 articles [79, 143, 178]	0 article
	Medical status	0 article	1 article [19]	1 article [98]
Social support	Low social support consistent	13 articles [9, 35, 37, 79, 143, 148, 177, 178, 179, 182, 185, 189, 194]	8 articles [58, 67, 79, 143, 148, 175, 178, 185]	1 article [81]
	Quality of life	2 articles [49, 192]	2 articles [19, 67]	0 article
	Living alone	Living alone: 4 articles [110, 162, 178, 195]	Living alone: 3 articles [162, 178, 195]	0 article
		Not living alone: 0 article	Not living alone: 0 article	0 article
	Less coping self-efficacy	1 article [42]	1 article [132]	0 article
	Violent	6 articles [13, 14, 16, 28, 70, 161]	1 article [70]	0 article
	Bullying	0 article	1 article [156]	0 article
	Incarceration	1 article [113]	1 article [113]	0 article
	Bereavement	2 articles [35, 79]	1 article [79]	0 article
Environment	Economic status inconsistent	Employment: 0 article	Employment: 2 articles [15, 61]	Employment: 1 article [105]
		Unemployment: 14 articles [9, 13, 18, 49, 50, 58, 75, 76, 142, 144, 147, 148, 164, 195]	Unemployment: 9 articles [19, 38, 124, 132, 147, 148, 162, 188, 195]	Unemployment: 3 articles [92, 98, 112]
	Race inconsistent	White: 4 articles [9, 40, 47, 49]	White: 1 article [47]	1 article [26]
		Black: 2 articles [49, 80]	Black: 0 article	Black: 0 article
	Religion	3 articles [142, 149, 192]	2 articles [19, 149]	0 article
	Having children inconsistent	Having children: 1 article [9]	Having children: 1 article [15]	Having children: 0 article
		Not having children: 0 article	Not having children: 0 article	Not having children: 1 article [115]
	Discrimination	1 article [110, 176]	0 article	0 article
Cause of death	Drug overdosage consistent	4 articles [28, 80, 82, 83]	2 articles [82, 83]	7 articles [17, 26, 30, 54, 81–83]
	Drug poisoning	0 articles	1 article [26]	1 article [26]
	Firearms consistent	1 article [28]	1 article [26]	1 article [26]
	Jumping	1 article [28]	1 article [26]	0 article
	Cutting wrists	1 article [28]	1 article [26]	0 article
	Suffocation.	0 article	1 article [26]	1 article [26]

Among the demographic factors, there were inconsistent risk factors for suicidal behavior such as gender (male [5, 33, 34, 81, 90, 111, 120], female [41, 92, 105]), age (young age [13, 16, 75, 77, 113, 138, 164], middle age[10, 18, 80, 142, 178], older age [9]), sexual orientation, education level, In respect to specific psychological symptoms and disorders, depression, substance abuse, anxiety, intravenous drug use, post-traumatic stress disorder, major mood disorders, and mental disorders were found to be consistent suicide risk factors [21, 22, 56, 65, 68, 74, 78, 97, 102, 116, 132, 135, 138, 141, 145–147, 149, 154, 155, 157, 158, 159, 172, 173, 154, 187, 192]. Among the physiological factors were HAART side effects, poor immune status, physical symptoms, comorbid illnesses, insomnia, CD4 cell count, unmonitored viral load, neurocognitive developmental disorders, opportunistic infections, and medical status; an inconsistent risk factor was HIV exposure time. The social factors of quality of life, living alone, less coping self-efficacy, violence, bullying, incarceration, and bereavement were inconsistent risk factors for suicidal behavior. However, low social support was a consistent risk factor. Among the environmental factors, socioeconomic status, ethnicity, and having children were inconsistent risk factors; however, discrimination and religion [142, 149, 193] were consistent risk factors (Table 2 and Fig 3).

### Measurement tools of the suicide behaviors suicidal behavior and risk factors

Within the included 193 studies, we found that 12 different scales were used to measured suicidal behavior and its risk factors; 26 studies used the Beck depression inventory scale, 8 used the Beck scale for suicide ideation, and 4 used the five-item brief symptom rating scale (Table 3).

**Table 3:** Measurement instrument of suicide.

Categories	Measurement tool	Studies
12 suicide scales	Beck depression inventory (BDI)	26
	[12, 18, 20, 24, 37, 46, 62, 85, 98, 126, 129–134, 137, 138, 164, 167, 169, 172, 176, 177, 180, 187]	
	Beck scale for suicide ideation (BSS) [16, 36, 46, 99, 175–177, 150]	8
	The five-item brief symptom rating scale (BSRS-5) [18, 159, 165, 166]	4
	Plutchik suicide risk scale [96]	1
	Positive and negative suicide ideation (PANSI) [51]	1
	GAIN scales: Suicidal/Homicidal thought scale [49]	1
	Harkavy Asnis Suicide Survey scale. [15]	1
	Suicide ideation self-report scale [36]	1
	Suicide ideation questionnaire (QIS) [16]	1
	Mini international psychiatric interview for children and adolescent’s suicidality and self-harm subscale [15]	1
	Suicide assessment questions from the national institute of mental health diagnostic interview schedule version III-A (DIS) [43]	1
	The electronic Columbia-suicidality severity rating scale (eC-SSRS) [69]	1

## Discussion

About 40 million people of the global population are currently living with HIV/AIDS. The era of HAART treatment has brought significant improvements in patient longevity and quality of life [202]; however, PLHIV experience a heavy burden of psychosocial conditions that are frequently undiagnosed and untreated. The pooled incidence of suicide completion among PLHIV globally was 10.2 per 1000 population, translating to a 100-fold greater suicide completion rate compared with the global population rate of 0.09/1000 population for 2019 [3, 203]. Therefore, this scoping review of 193 studies included an overview of three types of suicidal behavior among PLHIV as follows: suicidal ideation, suicidal attempts, and dying by suicides. We also included risk factors and associations of suicidal behavior according to demographic, social, physiological, psychological, and environmental factors. We identified consistent and inconsistent risk factors among the three types of suicidal behavior (Fig 3).

In total, this review encompasses 729,189 participants from 49 countries with all eligible articles published during the past 33 years (1988 to 2021). Two-thirds of the studies were published in the last five years (80/193). We found that there was an increasing trend toward conducting research related to suicidal behavior and risk factors among PLHIV globally.

Most studies were conducted in the United States or were performed by researchers from the United States conducting research in other countries, especially in Africa or developing countries (i.e., Nepal and Thailand). This is likely due to global funding strategies and continuing education programs conducted by United States universities along with partnership programs in other countries[14, 44, 75, 127, 129, 137, 138, 140–142, 144, 145, 154, 156, 157, 163, 186]. Most studies were conducted in hospitals and clinics. However, long-term observational data were extracted from databases as well, and of four studies conducted in prisons, three were in Taiwan [170, 171, 181], and one were in United States [73].

According to the findings of this review, the prevalence rate was highest in the United States, United Kingdom, Australia, and Russia for suicidal ideation, suicide attempts rate was highest in the United States, Australia, and Spain, and death due to suicide rate was highest in Denmark, and Thailand among PLHIV from 2000 to 2020. The highest suicide ideation rate was in the UK [84], followed by Australia [195], and the US [37]. The highest suicide attempt rate was in Australia [195], with the second highest in Spain [107], and third in South Africa [127]. The highest completed suicide rate was in Denmark [98], followed by Thailand [173], and France [112]. These findings may be since these countries have the most liberal laws on doctor-assisted suicide or gun control or could be due to economic recessions and societal pressure [26, 28, 204, 205]. These differences could be attributed to discrepancies in cultural differences, religious dimensions, and socioeconomic status, and not just by geographical location alone [4]. Previous research has identified psychological disorders and suicide are extremely connected and established in high-income countries, with many suicides occurring impulsively in moments of crisis with a breakdown in the ability to deal with life stresses. This review also found similar results[206, 207].

The most frequently used methods used of suicide are hanging and pesticide poisoning in Western countries[17, 26, 28, 30, 54, 80–83]. Reported risk factors for suicide attempts include mental and physical health problems, socioeconomic problems, and drug and alcohol use/abuse [208] According to our finding in when we considered about South-East Asian Region, most common suicide behavior is death due to suicide, compared with suicide attempts and suicide ideation. Because of educational status of family and social pressure also the social discrimination and stigma are more common in Asian countries than elsewhere in the world [206, 207].

Depression and suicidal thinking occur frequently alongside HIV/AIDS, triggering profound detrimental impacts on quality of life, treatment adherence, disease progression, and mortality [177, 209]. According to this scoping review, 85 articles dealing with depression, the most common death-related factor for PLHIV is suicide ideation, and their attempted suicide behavior risk is due to depression which is its common cause. Bullying, which includes stigmatization and discrimination, can also drive people to suicide as it increases social isolation. Substance abuse and overdose or severe physical disease are also recognized causes. According to this review findings, Caribbean countries and the Middle East showed the lowest death rates due to suicide.

This study is a global overview of suicidal behavior and associated risk factors among PLHIV. There are some important new findings in this review. First, our review provides both prevalence and incidence rates as well as risk factors for suicide ideation, suicide attempt, and death by suicide among PLHIV. Second, the current study includes findings from diverse populations of patients with HIV from 1988–2021, while previous reviews mostly focused on certain risk populations. Third, our study provides a group association and risk factors for suicidal ideation, suicide attempts, and death due to suicide. Therefore, we believe our findings suggest definite trends and factors that could prevent suicidal behavior among PLHIV, which future studies should examine further.

The limitations of this study were the lack of information regarding ethnic groups, cultural backgrounds, and religious perspectives of suicidal behavior and risk factors among PLHIV. Future studies should focus on these factors prospectively. Also, this large number of studies contained different type of confounding factors and it is difficult to control all confounding one time, however it will not influence to review findings because we would provide overview of suicidal behaviors only. Still did not make any causal relationship furthermore future study designed how to manage confounding such an incident if suicide actions. Also study quality is deferent to each study, not ranked study quality in terms of sample size, biases, etc. same as different scales/ measurement tools were used which also affects consistency in studies can consider some limitations.

## Conclusion

This scoping review presents a global view of suicidal behavior in 49 countries and included 193 primary research studies. We found that the Americas, Europe, and some Asia countries have the highest rates of suicidal behavior also after free access of antiviral therapy and post-HAART era, there has been an increasing trend in suicidal behavior. Depression, low quality of life, low social support, substance use, and drug abuse are the most common risk factors for suicidal behavior. Our study lacks information on ethnicity, cultural background, and religious perspectives of PLHIV, and those need to be considered in future studies. This review provides an overview of suicidal behavior and risk factors for future healthcare development plans and prevention of suicide in PLHIV.

### Clinical applications

This study will provide data on global suicidal ideation, suicide attempts, and completed suicide as well as the epidemiology and risk factors associated with completed suicides among people living with HIV. The findings of this review can be used as scientific evidence in the design of protocols and clinical practice guidelines intended to manage the wellbeing of PLHIV worldwide. It is also a reference for future researchers who plan to examine suicidal behavior and the risk factors among diverse populations. This study has practical implications for the management of people with HIV and preventing suicide at the global level. Given the high prevalence of suicide in high-risk populations such as people with HIV and the challenges related to preventing suicide, our study findings could support suicide prevention efforts by presenting the prevalence and incidence rates for suicide, as well as the associated risk factors among PLHIV.

## Supporting information

S1 Table Suicide ideation rate among people living with HIV. (DOCX)

S2 Table Suicide attempt rate among people living with HIV. (DOCX)

S3 Table Death due to suicide rate among people living with HIV. (DOCX)

S1 File SRSearchForm. (DOCX)

S1 Checklist (DOCX)
