# Supplementary material for: Global overview of suicidal behavior and associated risk factors among people living with human immunodeficiency virus: A scoping review
Source: PLoS One. 2023 Mar 20;18(3):e0269489. doi: 10.1371/journal.pone.0269489 (PMC10029973; doi:10.1371/journal.pone.0269489)
Supplement: S1 Table — (DOCX) [file pone.0269489.s001.docx]

# S1 Table. Suicide ideation rate among people living with HIV

| Years  Country | 1990 | 1993 | 1995 | 1996 | 1997 | 1998 | 2000 | 2002 | 2003 | 2004 | 2005 | 2006 | 2007 | 2008 | 2009 | 2010 | 2011 | 2012 | 2013 | 2014 | 2015 | 2016 | 2017 | 2018 | 2019 | 2020 | 2021 |
| --- | --- | --- | --- | --- | --- | --- | --- | --- | --- | --- | --- | --- | --- | --- | --- | --- | --- | --- | --- | --- | --- | --- | --- | --- | --- | --- | --- |
| Africa |  |  |  |  |  |  |  |  |  |  |  | 11.4%  [131] |  |  | 12%  [134] |  |  | 17.1%  [139]  24.2%  [142] | 5.9%  [143] | 28.8%  [146] | 24.3%  [23] | 39%  [15] | 4.1%  [163]  39%  [162] | 6.2%  [165] |  |  |  |
| Argentina |  |  |  |  |  |  |  |  |  |  |  |  |  |  |  |  |  |  |  |  |  |  |  | 35.6%  [79] | 21%  [81] |  |  |
| Australia |  |  |  |  |  |  |  |  |  |  |  |  |  |  |  |  |  |  |  | 67%  [202] |  |  |  |  |  |  |  |
| Brazil |  |  |  |  |  |  |  |  |  |  |  |  |  |  |  |  |  |  |  | 34.1%  [19]  50%  [60] |  |  |  |  |  |  |  |
| Canada |  |  |  |  |  |  |  |  |  |  |  |  |  |  |  |  | 9.2%  [54] |  |  |  |  |  | 22%  [73] |  |  |  |  |
| China |  |  |  |  |  |  |  |  |  |  |  |  |  |  |  | 34%  [174] | 49.3%  [177] |  |  |  | 31%  [183] |  | 25%  [185]  27.2%  [186] | 27.2%  [189]  31.6%  [11]  32.4%  [190] | 10.7%  [195] | 24.9%  [196] | 32.6%  [206] |
| Columbia |  |  |  |  |  |  |  |  |  |  |  |  |  |  |  |  |  |  |  |  |  |  |  | 14%  [75] |  |  |  |
| Estonia |  |  |  |  |  |  |  |  |  |  |  |  |  |  |  |  |  |  |  |  |  |  | 36%  [117] |  |  |  |  |
| Ethiopia |  |  |  |  |  |  |  |  |  |  |  |  |  |  |  |  |  |  |  |  |  | 33.6%  [147] | 22.5%  [154] |  |  | 24.3%  [167] | 8.2%  [205]  9.4%  [204]  16%  [203] |
| France |  |  |  |  |  |  |  |  | 13.2%  [90] |  |  | 18%  [93] |  |  |  |  |  | 1.28%  [103] |  |  |  |  | 6.3%  [114] |  |  |  |  |
| Greece |  |  |  |  |  |  |  |  |  |  |  |  |  |  |  |  |  |  |  |  |  |  |  |  | 9.2%  [123] |  |  |
| India |  |  |  |  |  |  |  |  |  |  |  |  |  |  |  |  |  |  | 12.3%  [181] |  |  |  |  |  |  |  |  |
| Indonesia |  |  |  |  |  |  |  |  |  |  |  |  |  |  |  |  |  |  |  |  |  |  |  |  |  | 23.3%  [12] |  |
| Iran |  |  |  |  |  |  |  |  |  |  |  |  |  |  |  |  |  |  |  |  |  |  |  | 15.4%  [164] |  |  |  |
| Malawi |  |  |  |  |  |  |  |  |  |  |  |  |  |  |  |  |  |  |  |  | 7.1%  [145] |  |  |  |  |  |  |
| Mexico |  |  |  |  |  |  |  |  |  |  |  |  |  |  |  |  |  |  |  |  |  | 10.4%  [71] |  |  |  |  |  |
| Nepal |  |  |  |  |  |  |  |  |  |  |  |  |  |  |  |  |  |  | 14%  [179] | 14%  [182] |  |  |  |  |  |  |  |
| Netherlands |  |  |  |  |  |  |  |  |  |  |  |  |  |  |  |  |  |  | 48%  [107] |  |  |  |  |  |  |  |  |
| Nigeria |  |  |  |  |  |  |  |  |  |  |  |  |  |  |  |  |  | 34.7%  [137] |  |  |  |  | 2.9%  [153]  15.1%  [157] |  |  | 35.3%  [166] |  |
| Romania |  |  |  |  |  |  |  |  |  |  |  |  |  |  |  |  | 12.1%  [100] |  |  |  |  |  |  |  |  |  |  |
| Russia |  |  |  |  |  |  |  |  |  |  |  |  |  |  |  |  |  | 56%  [106] |  |  |  |  |  |  |  |  |  |
| South Korea |  |  |  |  |  |  |  |  |  |  |  |  |  |  |  |  |  |  |  |  |  | 44%  [184] |  |  | 20%  [194] |  |  |
| Spain |  |  | 1.18%  [86] |  |  |  |  |  |  |  |  |  |  |  |  |  |  |  |  |  |  |  |  |  |  |  |  |
| Taiwan |  |  |  |  |  |  |  |  |  |  |  | 39.3%  [172] |  |  |  | 12.5%  [175] |  |  |  |  |  |  |  | 27.2%  [187]  [188] | 27.2%  [192]  45%  [191] |  |  |
| Tanzania |  |  |  |  |  |  |  |  |  |  |  |  |  |  |  |  |  |  |  |  |  |  |  | 10%  [164] |  | 12.8%  [168] |  |
| Uganda |  |  |  |  |  |  |  |  |  |  |  |  |  |  |  |  |  |  |  |  |  | 8.8%  [152]  10%  [151]  12.1%  [150] | 2.8%  [156]  3.3%  [155] | 6.2%  [161] |  |  |  |
| United  Kingdom |  |  | 50.5%  [87] |  | 69%  [88] |  |  |  |  |  |  |  |  |  |  |  |  |  |  |  |  | 56.6%  [113] |  | 16%  [120] |  |  |  |
| United States | 28.6%  [26] | 17%  [29]  32.5%  [200] |  | 55%  [201] | 12.8%  [36]  60%  [37] | 63.4%  [38] | 26%  [41] | 38%  [43] |  | 51.6%  [45] | 31%  [47] | 59.3%  [49] | 19%  [50] | 31%  [51] | 15%  [52] | 14%  [10] | 5.7%  [53] | 11%  [141]  26%  [22] | 28%  [59] | 7.3%  [59] |  |  | 8.6%  [74]  38%  [72] | 13%  [78] | 38.4%  [82] | 27.1%  [83] | 3.3%  [84] |
| Zimbabwe |  |  |  |  |  |  |  |  |  |  |  |  |  |  |  |  |  |  |  |  |  |  |  |  |  | 30.4%  [169] |  |
