# Supplementary material for: Global overview of suicidal behavior and associated risk factors among people living with human immunodeficiency virus: A scoping review
Source: PLoS One. 2023 Mar 20;18(3):e0269489. doi: 10.1371/journal.pone.0269489 (PMC10029973; doi:10.1371/journal.pone.0269489)
Supplement: S2 Table — (DOCX) [file pone.0269489.s002.docx]

# S2 Table. Suicide attempt rate among people living with HIV

| Years  Country | 1994 | 1995 | 1997 | 1998 | 2001 | 2003 | 2004 | 2005 | 2006 | 2008 | 2010 | 2011 | 2012 | 2013 | 2014 | 2015 | 2016 | 2017 | 2018 | 2019 | 2020 | 2021 |
| --- | --- | --- | --- | --- | --- | --- | --- | --- | --- | --- | --- | --- | --- | --- | --- | --- | --- | --- | --- | --- | --- | --- |
| Africa |  |  |  |  |  |  |  | 54%  [131] |  |  | 67.2Per  100,000  person  years  [135] |  |  | 5.9%  [143] |  |  |  | 6%  [158]  24%  [159] |  |  |  |  |
| Australia |  |  |  |  | 27%  [207] |  |  |  |  |  |  |  |  |  | 85.2%  [83] |  |  |  |  |  |  |  |
| Brazil |  |  |  |  |  |  |  |  |  |  |  |  |  |  |  |  | 18%  [68] |  |  |  |  |  |
| Canada |  |  |  |  |  |  |  |  |  |  |  | 10.3%  [54] |  |  |  |  |  | 5%  [73] |  |  |  |  |
| China |  |  |  |  |  |  |  |  |  |  | 8%  [174] |  |  | 43.1%  [180] |  |  |  |  | 9%  [190] | 3.2%  [195] |  | 12.2%  [206] |
| Columbia |  |  |  |  |  |  |  |  |  |  |  |  |  |  |  |  |  |  | 9%  [75] |  |  |  |
| Estonia |  |  |  |  |  |  |  |  |  |  |  |  |  |  |  |  |  | 20%  [117] |  |  |  |  |
| Ethiopia |  |  |  |  |  |  |  |  |  |  |  |  |  |  |  |  | 20.1%  [147] | 13.9%  [154] |  |  | 12.6%  [167] | 3.3%  [204]  7.1%  [203] |
| France |  |  |  |  |  |  |  |  |  | 22%  [128]  [129] |  |  |  |  |  |  |  |  |  |  |  |  |
| Greece |  |  |  |  |  |  |  |  |  |  |  |  |  |  |  |  |  |  |  | 14%  [123] |  |  |
| India |  |  |  |  |  |  |  |  |  |  |  |  |  |  |  |  |  |  |  | 23%  [193] |  |  |
| Nepal |  |  |  |  |  |  |  |  |  |  |  |  |  | 17%  [179] |  |  |  |  |  |  |  |  |
| Netherlands |  |  |  |  |  |  |  |  |  |  |  |  |  | 34%  [107] |  |  |  |  |  |  |  |  |
| Nigeria |  |  |  |  |  |  |  |  |  |  |  |  | 9.3%  [137] |  |  | 13.6%  [20] |  | 2.3%  [153]  3.9%  [157] |  |  |  |  |
| Puerto Rico |  |  |  |  |  |  |  |  |  |  |  |  |  |  | 20.4%  [64] |  |  |  |  |  |  |  |
| Russia |  |  |  |  |  |  |  |  |  |  |  |  | 36%  [106] |  |  |  |  |  |  |  |  |  |
| Rwanda |  |  |  |  |  |  |  |  |  |  |  |  |  |  |  |  | 12%  [149] |  |  |  |  |  |
| South  Korea |  |  |  |  |  |  |  |  |  |  |  |  |  |  |  |  | 11%  [184] |  |  |  |  |  |
| Spain |  | 4.02%  [170] |  |  |  |  |  |  |  |  |  |  |  |  |  | 72%  [111] |  |  |  |  |  |  |
| Taiwan |  |  |  |  |  |  |  |  | 26.7%  [172] |  | 4.1%  [175]  4.2%  [176] |  |  |  |  |  |  |  | 14%  [187]  14%  [188] | 9.7%  [191]  14.7%  [192] |  |  |
| Uganda |  |  |  |  |  |  |  | 17.4%  [132] |  |  |  | 5.9%  [136] | 3.9%  [140] |  |  |  | 3%  [151]  3.1%  [152]  6.2%  [150] |  | 13%  [160] |  |  |  |
| Ukraine |  |  |  |  |  |  |  |  |  |  |  |  |  |  |  |  |  |  |  |  | 28%  [126] |  |
| United  Kingdom |  | 21.4%  [87] | 31%  [88] |  |  |  |  |  |  |  | 27%  [97] |  | 6.6%  [105]  13%  [101]  34.7%  [104] |  |  |  |  |  |  |  |  |  |
| United  States | 21.8%  [30]  42.8%  [170] |  |  | 21%  [39]  52%  [40] |  | 44.3%  [44] | 25.8%  [45] | 16%  [47]  26%  [46] | 29.6%  [49] |  |  |  | 13%  [22] | 0.57%  [58]  23.7% [57] | 8.1%  [65]  31.5%  [61] |  | 35.3%  [70] |  |  | 16.9%  [83] |  |  |
