# Supplementary material for: Global overview of suicidal behavior and associated risk factors among people living with human immunodeficiency virus: A scoping review
Source: PLoS One. 2023 Mar 20;18(3):e0269489. doi: 10.1371/journal.pone.0269489 (PMC10029973; doi:10.1371/journal.pone.0269489)
Supplement: S3 Table — (DOCX) [file pone.0269489.s003.docx]

**S3 Table. Death due to suicide rate among people living with HIV**

| Years  Country | 1988 | 1992 | 1993 | 1994 | 1995 | 1996 | 1997 | 2002 | 2003 | 2004 | 2005 | 2006 | 2008 | 2010 | 2011 | 2012 | 2013 | 2014 | 2015 | 2016 | 2017 | 2018 | 2019 | 2020 |
| --- | --- | --- | --- | --- | --- | --- | --- | --- | --- | --- | --- | --- | --- | --- | --- | --- | --- | --- | --- | --- | --- | --- | --- | --- |
| Australia |  |  |  |  |  |  |  |  |  |  |  |  |  |  |  |  |  |  |  |  |  | 4%  [209] |  |  |
| Brazil |  |  |  |  |  |  |  | 0.5%  [42] |  |  |  |  |  |  |  |  |  |  |  |  |  |  |  |  |
| Canada |  |  |  | 9.4%  [130] |  |  |  |  |  |  | 7%  [48] |  |  | 2.81per 100,000 person years  [63] |  |  |  |  | 8.2%  [62] | 0.47  per  100 person-years  [67] |  |  |  |  |
| China |  |  |  |  |  |  |  |  |  |  |  | 4.8%  [171] |  |  |  |  |  |  |  |  |  |  |  |  |
| Denmark |  |  |  |  |  |  |  |  |  |  |  |  |  |  |  | 38.6%  [102] |  |  |  |  |  |  |  |  |
| France |  |  |  |  |  |  |  | 6%  [89] | 11%  [91] |  |  |  |  |  |  | 0.5%  [103] |  |  |  |  | 12.5%  [116] | 4.1%  [119] |  |  |
| Germany |  |  |  |  |  |  |  |  |  |  |  |  |  |  |  |  |  | 4%  [144] |  |  |  |  | 6.2%  [122] |  |
| Greece |  |  |  |  |  |  |  |  |  |  |  |  |  |  |  |  |  |  |  |  | 1.48%  [118] |  |  |  |
| Japan |  |  |  |  |  |  |  |  |  |  |  |  |  |  |  |  |  |  |  |  |  |  |  | 7.8%  [197] |
| Netherlands |  |  |  | 8.13%  [31] |  | 13%  [33] |  |  |  |  |  |  |  |  |  |  |  |  |  |  |  |  |  |  |
| Spain |  |  |  |  | 0.47%  [86] |  |  |  |  |  |  |  |  |  | 0.61%  [98] |  |  |  |  |  |  |  |  | 1.32%  [125] |
| Sweden |  | 25%  [85] |  |  |  |  |  |  |  |  |  |  |  |  |  |  |  |  |  |  |  |  |  |  |
| Switzerland |  |  |  |  |  |  |  |  |  |  |  |  |  | 158.4  per  100,000  person-  years  [5] |  |  | 6%  [108] |  | 0.4%  [109] |  |  |  | 1%  [124] |  |
| Taiwan |  |  |  |  |  |  |  |  |  |  |  |  | 5.5%  [173] |  |  |  |  |  |  |  |  |  |  |  |
| Thailand |  |  |  |  |  |  |  |  |  |  |  |  |  |  | 15.5%  [178] |  |  |  |  |  |  |  |  |  |
| United  Kingdom |  |  |  |  | 0.5%  [87] |  |  |  |  |  |  |  |  | 12.9%  [96] |  |  |  |  | 4.25  per 10,000 person-years  [110] | 0.23%  [112] | 2.1  per 10,000 person-years  [115] |  | 7%  [121] |  |
| United  States | 680.6  per  100,000 person-years  [25] | 167 per  100,000 person-years  [27] | 0.3%  [208] |  | 175 per 10,000person year  [32] | 49 per  100,000 person-years  [34] | 8.67%  [35] |  |  |  | 4%  [92] | 5%  [94] | 0.2%  [95]  5.5%  [51] |  |  | 1.5%  [56] |  |  |  |  |  | 7.6%  [76]  8%  [77] |  |  |
