# Supplementary material for: Global overview of suicidal behavior and associated risk factors among people living with human immunodeficiency virus: A scoping review
Source: PLoS One. 2023 Mar 20;18(3):e0269489. doi: 10.1371/journal.pone.0269489 (PMC10029973; doi:10.1371/journal.pone.0269489)
Supplement: S1 File — (DOCX) [file pone.0269489.s004.docx]

**Literature Search for Conducting Systematic Review: Documentation Form**

1. **PICO Question**

| **Question:** | What are the rates of suicidal behavior and associated suicide risk factors among PLHIV? |
| --- | --- |
| **P**opulation | Patient living with HIV/AIDS |
| **I**ntervention |  |
| **C**omparison |  |
| **O**utcome | Suicidal behavior |
| **T**ype of Question/  Publication **T**ype |  |

1. **Search Terms**

|  | Chinese Synonyms | English Synonyms | | **Emtree / MeSH**  Controlled Vocabulary |
| --- | --- | --- | --- | --- |
| **P** |  | | AIDS*  Human immunodeficiency virus*  Acquired immune deficiency syndrome virus*  T-lymphotropic virus*  Human T cell lymphotropic virus* type III  Human T cell leukemia virus* type III  Lymphadenopathy-associated virus*  LAV-HTLV-III  HTLV-III-LAV  Type III infection*  HTLV III infection* | Human immunodeficiency virus  Human immunodeficiency virus infection  acquired immune deficiency syndrome  HIV  HIV Infections  Acquired Immunodeficiency Syndrome |
| **I** |  | |  |  |
| **C** |  |  | |  |
| **o** |  | suicid*  automutilat*  auto mutilat*  head bang*  selfcut*  selfdeliberat*  selfdestruct*  selfharm*  selfimmolat  selfinflict*  selfinjur*  selfkill*  selfmutilat*  selfpoison*  selfwound*  self  NEAR/2  cut  cutting  deliberat*  destruct*  harm*  immolat  inflict*  injur*  kill*  mutilat*  poison*  wound* | | Suicidal behaviour  suicide  Automutilation  [Self-Injurious Behavior](https://www.ncbi.nlm.nih.gov/mesh/68016728) |

3. **Searched Databases**

| **Database** | | **Date searched** | | **Citations** | | | | | | |
| --- | --- | --- | --- | --- | --- | --- | --- | --- | --- | --- |
|  |  |  |  | **Records identified through database searching** | | **Records after duplicates removed / Records screened** | | **Full-text articles assessed for eligibility** | **Studies included in qualitative synthesis** | **Studies included in quantitative synthesis** |
| 1. Embase | | 20210713 | | 2,247 | | **included: 4925** | | **included:  1958** | **included:  193** | **included: 0** |
| 1. MEDLINE | | 20210713 | | 916 | |  |  |  |  |  |
| 1. CENTRAL | | 20210713 | | 863 | |  |  |  |  |  |
| 1. Web of science | | 20210713 | | 2,222 | |  |  |  |  |  |
| 1. Academic Search Complete | | 20210715 | | 1,503 | |  |  |  |  |  |
| 1. Psychology Behavioral Sciences | | 20210715 | | 335 | |  |  |  |  |  |
| additional sources: manual search | | 20210715 | | 44 | |  |  |  |  |  |
| **included: 8130** |  | | |  | | |  |  | | |
| **de-duplicates:**  **3205** | | | excluded:  2967  1,736 theses  849 no information  156 reviews  121 case studies 105 qualitative studies | | excluded:  1765  1,310 unrelated to HIV  357 unrelated to suicide  52 had no relevant data  46 no full text | | | **excluded:** | | |

**4. Search Strategy**

| **Database** |  | **Search syntax** | **Citations found** |
| --- | --- | --- | --- |
| 1. **Embase**   [**(Elsevier)**](javascript:;) | **1** | **(AIDS OR Human immune deficiency virus* OR Acquired immune deficiency syndrome virus* OR T-lymphotropic virus* OR Human T cell lymphotropic virus* type III OR Human T cell leukemia virus* type III OR Lymphadenopathy-associated virus* OR LAV-HTLV-III OR HTLV-III-LAV OR Type III infection* OR HTLV III infection*):ti,ab,kw,de** | **17,795** |
|  | **2** | **"Human immunodeficiency virus"/exp OR "Human immunodeficiency virus infection"/exp** | **509,270** |
|  | **3** | **(suicid* OR automutilat* OR auto mutilat* OR head bang* OR selfcut* OR selfdeliberat* OR selfdestruct* OR selfharm* OR selfimmolat OR selfinflict* OR selfinjur* OR selfkill* OR selfmutilat* OR selfpoison* OR selfwound* OR self NEAR/2 (cut OR cutting OR deliberat* OR destruct* OR harm* OR immolat OR inflict* OR injur* OR kill* OR mutilat* OR poison* OR wound*)):ti,ab,kw,de** | **28,069** |
|  | **4** | **"suicidal behavior"/exp OR "automutilation"/exp** | **128,529** |
|  | **5** | **1 OR 2** | **525,140** |
|  | **6** | **3 OR 4** | **137,611** |
|  | **7** | **5 AND 6** | **2,247** |

| 1. **MEDLINE**   **(OVID)** | **1** | **(AIDS OR Human immune deficiency virus* OR Acquired immune deficiency syndrome virus* OR T-lymphotropic virus* OR Human T cell lymphotropic virus* type III OR Human T cell leukemia virus* type III OR Lymphadenopathy-associated virus* OR LAV-HTLV-III OR HTLV-III-LAV OR Type III infection* OR HTLV III infection*).mp** | **211,613** |
| --- | --- | --- | --- |
|  | **2** | **exp "HIV Infections"/ OR exp "HIV"/** | **329,928** |
|  | **3** | **(suicid* OR automutilat* OR auto mutilat* OR head bang* OR selfcut* OR selfdeliberat* OR selfdestruct* OR selfharm* OR selfimmolat OR selfinflict* OR selfinjur* OR selfkill* OR selfmutilat* OR selfpoison* OR selfwound* OR self ADJ2 (cut OR cutting OR deliberat* OR destruct* OR harm* OR immolat OR inflict* OR injur* OR kill* OR mutilat* OR poison* OR wound*)).mp** | **25,917** |
|  | **4** | **exp "Self-Injurious Behavior"/** | **75,580** |
|  | **5** | **1 OR 2** | **415,833** |
|  | **6** | **3 OR 4** | **86,048** |
|  | **7** | **5 AND 6** | **916** |

| 1. **CENTRAL**   **(EBSCOhost)** | **1** | **AIDS OR Human immune deficiency virus* OR Acquired immune deficiency syndrome virus* OR T-lymphotropic virus* OR Human T cell lymphotropic virus* type III OR Human T cell leukemia virus* type III OR Lymphadenopathy-associated virus* OR LAV-HTLV-III OR HTLV-III-LAV OR Type III infection* OR HTLV III infection*** | **134,637** |
| --- | --- | --- | --- |
|  | **2** | **HIV Infections OR HIV** | **120,815** |
|  | **3** | **suicid* OR automutilat* OR auto mutilat* OR head bang* OR selfcut* OR selfdeliberat* OR selfdestruct* OR selfharm* OR selfimmolat OR selfinflict* OR self-insure* OR selfkill* OR selfmutilat* OR selfpoison* OR selfwound* OR self ADJ2 (cut OR cutting OR deliberat* OR destruct* OR harm* OR immolat OR inflict* OR injur* OR kill* OR mutilat* OR poison* OR wound*)** | **45,617** |
|  | **4** | **MH(Self-Injurious Behavior+)** | **4,515** |
|  | **5** | **1 OR 2** | **157,311** |
|  | **6** | **3 OR 4** | **48,132** |
|  | **7** | **5 AND 6** | **863** |

| 1. **Web of science** | **1** | **AIDS OR Human immune deficiency virus* OR Acquired immune deficiency syndrome virus* OR T-lymphotropic virus* OR Human T cell lymphotropic virus* type III OR Human T cell leukemia virus* type III OR Lymphadenopathy-associated virus* OR LAV-HTLV-III OR HTLV-III-LAV OR Type III infection* OR HTLV III infection*** | **661,148** |
| --- | --- | --- | --- |
|  | **2** | **HIV Infections OR HIV** | **408,054** |
|  | **3** | **suicid* OR automutilat* OR auto mutilat* OR head bang* OR selfcut* OR selfdeliberat* OR selfdestruct* OR selfharm* OR selfimmolat OR selfinflict* OR selfinjur* OR selfkill* OR selfmutilat* OR selfpoison* OR selfwound* OR self ADJ2 (cut OR cutting OR deliberat* OR destruct* OR harm* OR immolat OR inflict* OR injur* OR kill* OR mutilat* OR poison* OR wound*)** | **118,448** |
|  | **4** | **Self-Injurious Behavior** | **3,991** |
|  | **5** | **#2 OR #1** | **661,393** |
|  | **6** | **#4 OR #3** | **665,093** |
|  | **7** | **#6 AND #5** | **2,222** |

| 1. **Academic Search Complete (EBSCOhost)** | **1** | **AIDS OR Human immune deficiency virus* OR Acquired immune deficiency syndrome virus* OR T-lymphotropic virus* OR Human T cell lymphotropic virus* type III OR Human T cell leukemia virus* type III OR Lymphadenopathy-associated virus* OR LAV-HTLV-III OR HTLV-III-LAV OR Type III infection* OR HTLV III infection*** | **316,933** |
| --- | --- | --- | --- |
|  | **2** | **HIV Infections OR HIV** | **216,513** |
|  | **3** | **suicid* OR automutilat* OR auto mutilat* OR head bang* OR selfcut* OR selfdeliberat* OR selfdestruct* OR selfharm* OR selfimmolat OR selfinflict* OR self-insure* OR selfkill* OR selfmutilat* OR selfpoison* OR selfwound* OR self ADJ2 (cut OR cutting OR deliberat* OR destruct* OR harm* OR immolat OR inflict* OR injur* OR kill* OR mutilat* OR poison* OR wound*)** | **101,853** |
|  | **4** | **MH(Self-Injurious Behavior+)** | **6,724** |
|  | **5** | **1 OR 2** | **348,388** |
|  | **6** | **3 OR 4** | **107,050** |
|  | **7** | **5 AND 6** | **1,503** |

| 1. **Psychology Behavioral Sciences** | **1** | **AIDS OR Human immune deficiency virus* OR Acquired immune deficiency syndrome virus* OR T-lymphotropic virus* OR Human T cell lymphotropic virus* type III OR Human T cell leukemia virus* type III OR Lymphadenopathy-associated virus* OR LAV-HTLV-III OR HTLV-III-LAV OR Type III infection* OR HTLV III infection*** | **29,465** |
| --- | --- | --- | --- |
|  | **2** | **HIV Infections OR HIV** | **23,463** |
|  | **3** | **suicid* OR automutilat* OR auto mutilat* OR head bang* OR selfcut* OR selfdeliberat* OR selfdestruct* OR selfharm* OR selfimmolat OR selfinflict* OR self-insure* OR selfkill* OR selfmutilat* OR selfpoison* OR selfwound* OR self ADJ2 (cut OR cutting OR deliberat* OR destruct* OR harm* OR immolat OR inflict* OR injur* OR kill* OR mutilat* OR poison* OR wound*)** | **15,198** |
|  | **4** | **MH(Self-Injurious Behavior+)** | **1,911** |
|  | **5** | **1 OR 2** | **32,598** |
|  | **6** | **3 OR 4** | **15,268** |
|  | **7** | **5 AND 6** | **335** |
