# Supplementary material for: Global overview of suicidal behavior and associated risk factors among people living with human immunodeficiency virus: A scoping review
Source: PLoS One. 2023 Mar 20;18(3):e0269489. doi: 10.1371/journal.pone.0269489 (PMC10029973; doi:10.1371/journal.pone.0269489)
Supplement: Attachment — Submitted filename: Reply to reviewers(1).docx [file pone.0269489.s006.docx]

E-mail: nyko@mail.ncku.edu.tw

Tel: 886-6-2353535 ext. 5838

Fax: 886-6-2277550

國 立 成 功 大 學 醫 學 院

NATIONAL CHANG KUNG UNIVERSITY

護理系	柯乃熒

**Nai-Ying Ko, RN., Ph.D.**

*Department of Nursing,*

*College of Medicine,*

*National Cheng Kung University*

**No.1, Ta-Hsueh Road, Tainan 701, Taiwan.**

**台灣 台南市大學路一號**

**Journal of PLOS ONE**

Saeed Ahmed, PhD
Editor-in-Chief

RE: [PONE-D-21-32836] - [EMID:5107eee60dc38229]

Title: Global overview of suicidal behavior and associated risk factors among people living with human immunodeficiency virus: a scoping review

Dear Professors, Saeed Ahmed,

We highly appreciate your constructive comments and valuable suggestions to our manuscript. We have edited this manuscript according to your journal additional requirements and have revised our manuscript highlight with red colour according to all reviewers’ suggestions, and explain the changes as noted below:

**Responses to** **Reviewers:**

**Reviewer #1:**

1. This systematic review reviewing global literature to document suicidal behavior and risk factors among people living with HIV from Jan 1 1988 to July 8 2021. The purpose is to help prevent suicidal risk among PLHIV. It is an original literature review. With such a wide-ranging study, it can be difficult to find consistent findings or learning points especially with various confounding factors in various studies which are difficult to adjust for A number of risk factors and suicidal behavioral patterns were observed among these papers. The selection of publications to review was extensive which could lead various inconsistent conclusions in addition to few consistent ones including psychiatric conditions, low social support, discrimination. As per authors, previous primary research had shown poor social support, HIV stigma, mental disorders and associated co-morbidities as risk factors.
   Clearly, the authors have worked hard and gone through a lot of papers for this manuscript. It was quite interesting and informative to read. I have a few comments/ questions about it that I believe could help make it more suitable for publication. This includes minor grammatical errors to major questions.
(1) Authors have mentioned several times (e.g.- Line 32, line 204 and also in conclusion) that suicide rates have increased from the pre-HAART era to Post HAART recently. This seemed unlikely to me and looked into citations you have made. Citation 5 says suicide decreased to 0. And in citation 63, it says suicide proportion has increased as aids related cause of death has decreased. Citation 4 mentions "Suicide rates decreased significantly with the introduction of HAART". Please correct me with evidence that completed suicides have increased since HAART was brought in.

**Response**: Thank you for the comments. We have changed and added new citation as “The rate of suicide deaths in People living with HIV (PLHIV) is 100-fold higher than the rate that has been reported in the general population[3]. Prevalence estimates of suicidal ideation, attempts, and plans among people living with HIV/AIDS were more common and serious than those in the general population[4]. Suicide attempt rates among PLHIV with mental disorders and psychiatric treatment have continued to increase from the pre-highly active antiretroviral therapy (Pre-HAART) era (1988–1995) to the HAART era (1996–2008) from 27.8% to 35.1%, respectively[5]. Please refer the introduction section and line no 56 to 63 and Reference no 3, 4, 5.

(2) If you are stating that suicidal rates have in fact increased in this time, could you mention why that is? Which risk factors have worsened? when you say suicide rates have increased, do you mean suicides are causing higher proportion of deaths because people are living longer due to HAART and not dying due to AIDS as much? If so, that needs clarifying.

**Response**: Thank you for the suggestions and have added as “Suicide attempt rates among PLHIV with mental disorders and psychiatric treatment have continued to increase from the pre-highly active antiretroviral therapy (Pre-HAART) era (1988–1995) to the HAART era (1996–2008) from 27.8% to 35.1%, respectively[5]. Please refer the introduction section and line no 60 to 63 and Reference no 5.

(3) Line 28- 800k died every year - in which years or is that an average since 1988? Please clarify.

**Response:** Thank you for comments. We changed as “approximately 700,000 people died worldwide due to suicide every year [1].” Please refer introduction section and line no 50 to 52.

(4) Line 50-53: Review sentence grammar.

**Response:** Thank you for the comment. We corrected grammar in this manuscript using scribendi.com for this time.

(5) Line 47: Relation between 3 suicide behaviors- It would be interesting if you could specify certain risk factors that are more associated with either one of these 3 suicidal behaviors separately or other different relationships between the 3. Just a suggestion.

**Response:** Thank you for the suggestions and we simply changed as per your suggestion like this “In the current study provided insights into the relationships among HARRT, depression, and suicidal status in PLHIV and evidence that depression played a mediating role in the association between suicide ideation and attempt. However, the relationship between these three-suicide behavior is unclear; for example, relationship between HARRT, and death by suicide or depression, and suicide attempts, therefore, this study will be a better feasibility to understanding relationship between these three-suicide behavior.” Please refer introduction in line number 75 to 81.

(6) Line 165- Pre* HAART not Per*.

**Response:** Thank you for the correction. We changed as “Pre” please refer result section and line no 204.

(7) Line 225- Some risk factors are confusing such as 'religion', 'gender', 'age'. Could you specify what the papers say about how these factors affect risk? Which ages, genders, etc?

**Response:** Thank you for your suggestions. We have changed as “gender (male [4, 34, 35, 84, 93, 114, 122], female [42, 95, 108]), age (young age [3, 60, 78, 80, 140, 160, 167], middle age [10, 19, 83, 144, 182], older age [11]), and religion [144, 151, 197].” Please refer result section and line 252 to 253 and 267.

(8) Discussion- Line 253-255- "Suicide prevalence highest in Americas/ Europe from 1997 to 2020" but then you list countries that has Australia, Thailand, South Africa as top prevalence in the next few lines.

**Response**: Thank you for the comments. We changed as “United States, United Kingdom, Australia, and Russia for suicidal ideation, suicide attempts rate was highest in the United States, Australia, and Spain, and death due to suicide rate was highest in Denmark, and Thailand among PLHIV from 2000 to 2020.” Please see the discussion section line 297 t0 299.

(9) Discussion- Line 259-Line 261: You mentioned reasons as liberal laws on doctor-assistant suicide, gun control or economic/ social pressures: Any citations for that?

**Response:** Thank you for the suggestions. We added these citations [27, 29, 209, 210].

(10) Discussion- Line 268- Line 270- "85% of suicides occur in Africa and middle/low-income countries": Citation?

**Response:** Thank you for the suggestions. We simply removed that sentence because it has some typo missing and error information.

(11) You could mention higher risk of suicide in people with HIV compared to general population to emphasize suicidal risks in PLHIV (as seen in citations you have provided).

**Response:** Thank you for the suggestions. We have changed our introductions as “The rate of suicide deaths in People living with HIV (PLHIV) is 100-fold higher than the rate that has been reported in the general population[3].” and added one reference (Ref: No 3). Also, in the discussion we started as “About 40 million people of the global population are currently living with HIV/AIDS. The era of HAART treatment has brought significant improvements in patient longevity and quality of life; however, PLHIV experience a heavy burden of psychosocial conditions that are frequently undiagnosed and untreated. The pooled incidence of suicide completion among PLHIV globally was 10.2 per 1000 population, translating to a 100-fold greater suicide completion rate compared with the global population rate of 0.09/1000 population for 2019[3, 210]. With two citations. Please refer line 56 to 57 in introduction and line 276 to 282 in discussion.

(12) Discussion- Line 274- In Asian region, most common suicide behavior is completed suicide. So increased completed suicides compared to other regions (that goes against previous report that suicidal behaviors highest in Americas/ Europe) or completed suicides are higher than suicidal ideation/ attempts (That's not possible).

**Response**: Thank you for the comments. We changed as “According to our finding in when we considered about South-East Asian Region, most common suicide behaviour is death due to suicide, compared with suicide attempts and suicide ideation. Because of educational status of family and social pressure also the social discrimination and stigma are more common in Asian countries than elsewhere in the world [213, 214]. Please refer line 323 to 327 in discussion section. Send for English editing as well.

(13) Discussion- Line 278 - "Most common death factor?"; "Depression and its common cause." Please correct that whole sentence to be clear grammatically.

**Response**: Thank you for the suggestion. We change as “Depression and suicidal thinking occur frequently alongside HIV/AIDS, triggering profound detrimental impacts on quality of life, treatment adherence, disease progression, and mortality[182, 216]. According to this scoping review, 85 articles dealing with depression, the most common death-related factor for PLHIV is suicide ideation, and their attempted suicide behavior risk is due to depression which is its common cause.” Please refer line328 to 333 in discussion.

(14) In Discussion- It would help to mention something the reader should take from this study that could help clinically such as importance of social supports, etc in HIV treatment.

**Response:** Thank you for the suggestions: we added subtopic of clinical applications as “The findings of this review can be used as scientific evidence in the design of protocols and clinical practice guidelines intended to manage the wellbeing of PLHIV worldwide. It is also a reference for future researchers who plan to examine suicidal behavior and the risk factors among diverse populations. This study has practical implications for the management of people with HIV and preventing suicide at the global level. Given the high prevalence of suicide in high-risk populations such as people with HIV and the challenges related to preventing suicide, our study findings could support suicide prevention efforts by presenting the prevalence and incidence rates for suicide, as well as the associated risk factors among PLHIV.” Please prefer line no 373 to 382 in clinical application section.

(15) Conclusion- "We found that Americas, Europe and Asia have the highest rates of suicidal behavior" - Compared to what? Africa and Australia? Better if conclusion is more specific. Please change the wording to explain what you mean.

**Response:** Thank you for the suggestions. We change as “We found that the Americas, Europe, and some Asia countries have the highest rates of suicidal behavior also after free access of antiviral therapy and post-HAART era, there has been an increasing trend in suicidal behavior.” Please prefer line No 361 to 364 in conclusion.

(16) How did your results compare to what we knew from previous research studies- Confirmed some? Expanded any additional information we didn't know before? Add them in conclusion as important findings from this study.

**Response**: Thank you for the suggestions. We added one section as a clinical application as “The findings of this review can be used as scientific evidence in the design of protocols and clinical practice guidelines intended to manage the wellbeing of PLHIV worldwide. It is also a reference for future researchers who plan to examine suicidal behavior and the risk factors among diverse populations. This study has practical implications for the management of people with HIV and preventing suicide at the global level. Given the high prevalence of suicide in high-risk populations such as people with HIV and the challenges related to preventing suicide, our study findings could support suicide prevention efforts by presenting the prevalence and incidence rates for suicide, as well as the associated risk factors among PLHIV.” Please refer line No: 373 to 382 in clinical application section.

(17) The figures and tables are well done. Would help to have a table with consistent/ inconsistent risk factors.

**Response:** Thank you for the comments. We have added some new data to table 01 and table 02 contained data of consistent/ inconsistent risk factors.

(18) There are several limitations you can add at the end.

Different types of studies can have different qualities of study. Not evaluating which studies are better in terms of sample size, biases, etc. is a limitation.

Given different time periods, cultures etc, in regional studies review, it is difficult to make generalized conclusions for global factors and patterns. Recent studies more valuable than pre-HAART studies.

Different scales/ measurement tools were used which also affects consistency in studies.

**Response:** Thank you for the suggestions. We changed our limitations as “Also, this large number of studies contained different type of confounding factors and it is difficult to control all confounding one time, however it will not influence to review findings because we would provide overview of suicidal behaviors only. Still did not make any causal relationship furthermore future study designed how to manage confounding such an incident if suicide actions. Also study quality is deferent to each study, not ranked study quality in terms of sample size, biases, etc. same as different scales/ measurement tools were used which also affects consistency in studies can consider some limitations”. Please refer line No: 348 to 355 in limitation section.

(19) Many grammatical errors and spelling errors noted in the manuscript. It would be helpful to review it once more.

**Response:** Thank you for the suggestions. We have sent this manuscript for English editing by scribendi.com for this time.

**Reviewer #2:**

Some of the sentences are repetitive specially definitions of different suicidal behaviours including ideations and attempts and can be revised. Good description of consistent and inconsistent risk factors.

**Response:** Thank you for your great feedback.

**Reviewer #3:**

The submitted manuscript discusses an important topic of suicide in patients with HIV. However, in its current form, the article suffers from some deficiencies which need to be addressed. A lot of focus has been placed on tabular display of data rather than the written sections. The introduction section especially has been presented in a very matter of fact fashion and does not evoke much curiosity in the reader. The authors need to cite more articles to drive home the point that suicide in patients with HIV is a matter of public health concern. It would be helpful to tie in Hypersexual behaviours, sexual addiction, and other psychiatric disorders to HIV.
I refer the authors to the following article which discussed hypersexuality and sexual addiction to risk of getting STDs. Kindly review and cite the article as appropriate.
[https://www.researchgate.net/profile/Ashish-Sarangi 2/publication/320741412_Hypersexual_Disorder-A_Case_Report_and_Analysis_A_R_T_I_C_L_E_I_N_F_O/links/59f916ea0f7e9b553ec0c8ec/Hypersexual-Disorder-A-Case-Report-and-Analysis-A-R-T-I-C-L-E-I-N-F-O.pdf](https://www.researchgate.net/profile/Ashish-Sarangi-2/publication/320741412_Hypersexual_Disorder-A_Case_Report_and_Analysis_A_R_T_I_C_L_E_I_N_F_O/links/59f916ea0f7e9b553ec0c8ec/Hypersexual-Disorder-A-Case-Report-and-Analysis-A-R-T-I-C-L-E-I-N-F-O.pdf)
Although not the primary aim, it would be helpful to include a few lines in the discussion section making suggestions on how to screen patients at risk of suicide and refer for appropriate interventions.
It will also be helpful to discuss the main methods used for suicide if this data is available for example hanging e.t.c
Please condense the tables as there is too much data in the tables for meaningful review.
I will be happy to accept a revised manuscript for consideration as I believe it has potential.

**Response**: Thank you for the comments and suggestions. We cited this article in discussion and changed some table and figure data again also the rewrite some area in introduction, results and discussion as per your suggestions. Please refer line No: 56 to 81, and No: 275 to 355 in limitation section.

**Reviewer #4:**

Thank you for the opportunity to review the article.
The review is an excellent and balanced overview that includes 193 observational studies, a large sample size from 49 countries, both clinic and hospital data from multiple databases, encompasses diverse populations, 23 years of data( 2/3 of papers were recent), gives an interesting perspective on historical pre HAART suicide rate compared to post HAART suicide rate in HIV patient that ironically showed an increasing trend of suicidal behavior post-HAART therapy which is essential to be addressed and is often overlooked. Furthermore, they have highlighted risk factors of suicidal ideation and complete suicide in detail in different countries (included physical, psychological, social, demographic risk factors), also used standard scales as their guide, talked about methods of committing suicide in different countries, information on prevalence, association with certain coexisting conditions like substance disorder, mental illness, etc. They have registered in INPLASY, has Prisma flow diagrams, appropriate methodology for review and bias, did JBI for ROB assessment, and have good review article tables and flow charts.
However, I have few suggestions that could be added to the article.
In my opinion, a key feature that could be included in the article is, using all patient sample data from various articles divided into baseline characteristics such as age, sex, ethnicity, family history data, cultural and social aspects, important comorbid conditions like substance use disorder, stigma, mental illness, and side effects of any HAART therapy causing suicidal ideation. Then, subsequently analysed the total patient sample to derive any associations and protective factors. Please elaborate more on the third point mentioned in the discussion part and, if able to, try to describe any methods that can tackle this situation of suicide in post HAART HIV patients with a positive angle. Language is unclear at certain parts of the papers, making it difficult to follow. Therefore, I advise the authors to revise with Minor phrasing and grammatical issues to improve the flow and readability of the text.

**Response:** Thanks for your valuable comments and suggestions. We have rechecked all the data again and updated table and figure. Also write rewrite some section in introductions, result and discussions also modified limitations. Added one sections for clinical applications. Please refer line No: 56 to 81, and No: 275 to 355 in limitation section.
